# The effects of valine, lysine and threonine and their optimal combinations on the synthesis of α-casein by MAC-T cells

**DOI:** 10.3389/fvets.2026.1780614

**Published:** 2026-03-18

**Authors:** Min Yang, Xinyu Zhang, Yu Ding, Lewen Xie, Yu Gao, Kangyu Yao, Wanping Ren, Liang Yang, Yankun Zhao, Wei Shao

**Affiliations:** 1 Xinjiang Key Laboratory of Meat and Milk Production Herbivore Nutrition, College of Animal Science, Xinjiang Agricultural University, Urumqi, China; 2 Xinjiang Academy of Agricultural Sciences, Xinjiang Uygur Autonomous Region, Institute of Agricultural Quality Standards and Testing Technology, Agricultural Products Quality and Safety Risk Assessment Laboratory of the Ministry of Agriculture and Rural Affairs (Urumqi), Xinjiang Agricultural Products Quality and Safety Laboratory, Urumqi, China

**Keywords:** lysine, MAC-T, mTOR signaling pathway, the optimal combination of amino acids, threonine, valine

## Abstract

This study aims to enhance milk production and quality in dairy cows. Using *in vitro* cultured MAC-T cells as a model, it seeks to investigate the effects of valine, lysine, and threonine, as well as their optimal combinations, on the synthesis of α-casein by MAC-T cells. Following a 12-h serum starvation period, MAC-T cells were supplemented with varying concentrations of each amino acid individually. The appropriate concentration ranges and optimal levels for valine, lysine, and threonine were determined using ELISA. Response surface methodology was employed to identify the optimal combination of the three amino acids. The resulting α-casein synthesis in the combined treatment group (MIX group) was then compared with that achieved at the individual optimal concentrations and validated by ELISA. Furthermore, mRNA expression levels of the α-casein-encoding gene and key components of the mTOR signaling pathway were analyzed by RT-qPCR, while protein phosphorylation levels were assessed via Western blot. To confirm the functional involvement of the mTOR pathway, a rapamycin-based inhibition assay was conducted. The maximal stimulation of α-casein synthesis in MAC-T cells was observed at valine, lysine, and threonine concentrations of 4 × Val (25.528 mmol/L), 1 × Lys (7.364 mmol/L), and 0.5 × Thr (1.473 mmol/L), respectively. The optimal amino acid combination (MIX) was determined to be valine:lysine:threonine = 36.114:9.027:4.602 mmol/L. α-Casein synthesis in the MIX group was significantly higher than in any individual amino acid supplementation group (*p* < 0.01). Supplementation with the MIX medium markedly upregulated the relative mRNA expression of α-casein encoding genes (*CSN1S1* and *CSN1S2*) and key components of the mTOR signaling pathway (*ragA-D*, *mTOR*, *MLST8*, *RPTOR*, *EIF4EBP1*, *EIF4E*, *S6K1*, *EEF2*, and *RPS6*), as well as enhanced the phosphorylation levels of mTOR pathway-related proteins (mTOR, S6K1, 4EBP1, RPS6, and eEF2) (*p* < 0.01). Treatment with rapamycin significantly suppressed the mRNA expression of these genes, reduced protein phosphorylation, and inhibited α-casein synthesis (*p* < 0.01); however, co-supplementation with the optimal amino acid combination partially alleviated this suppression, indicating a protective regulatory role of the MIX formulation. The optimal combination of valine, lysine, and threonine was determined to be 36.114:9.027:4.602 mmol/L, corresponding to an approximate molar ratio of 8:2:1. This specific ratio significantly promotes α-casein synthesis in MAC-T cells through activation of the mTOR signaling pathway.

## 1 Introduction

Valine, lysine and threonine are all essential amino acids for mammals (cattle). Compared with other amino acids, they are among the most abundant amino acids in the α-casein component. For instance, in bovine αs1-casein, lysine, threonine and valine account for 7.0, 2.8, and 6.1% of its 214 amino acid residues respectively; in αs2-casein, they account for 11.3, 7.2, and 6.8% of its 222 amino acid residues, respectively. The high proportion of these amino acids in the α-casein polypeptide chain reflects their significant potential as substrates for α-casein synthesis and in determining the synthesis quantity of α-casein. Besides their role as protein synthesis substrates, these amino acids also function as signaling molecules. Valine is not only an important component of casein but also acts as a signaling molecule to promote casein synthesis by activating the mTOR pathway (1); lysine is the first limiting amino acid for casein synthesis, and its deficiency significantly reduces the amount of casein synthesized (2); threonine is a key functional amino acid for maintaining the homeostasis of casein synthesis, and its deficiency leads to premature termination or low efficiency of peptide chain synthesis (3). The synthesis of casein is regulated by multiple amino acids in concert. Its synthesis efficiency depends on the coordinated supply and precise balance of various amino acids. The supplementation of a single amino acid often fails to achieve the optimal effect (4). Valine, lysine and threonine have a high proportion in the polypeptide chain of α-casein and act as signal molecules to promote the synthesis of α-casein. It is precisely because of this dual importance that they have become the focus of this study. Therefore, it is highly significant to systematically investigate the effects and underlying regulatory mechanisms of the optimal combination of valine, lysine, and threonine on casein synthesis in bovine mammary epithelial cells. Previous studies have demonstrated that supplementing MAC-T cell culture medium with individually optimized levels of valine, lysine, or threonine significantly enhances intracellular casein synthesis; however, excessive supply of these amino acids may suppress both the quantity and rate of casein synthesis (5, 6). Gao et al. (7) demonstrated that supplementing the culture medium of primary bovine mammary epithelial cells with histidine, lysine, methionine, and leucine at a ratio of 5:6:1:7 significantly enhanced intracellular β-casein synthesis, reaching maximal levels through activation of the mTOR signaling pathway. Liu et al. (8) reported that supplementing the culture medium of *in vitro*-cultured bovine mammary epithelial cells with lysine and methionine at a ratio of 3:1 led to maximal expression of αs1-casein mRNA. Currently, studies have shown that supplementing valine, lysine, or threonine individually can promote α-casein synthesis in MAC-T cells; however, the effects are dose-dependent and fail to achieve maximal synthetic efficiency. The single amino acid paradigm is no longer fully sufficient to explain casein synthesis in lactating dairy cows. Coordinated supply of multiple amino acids, precise molar ratios, and activation of key signaling pathways are now recognized as critical factors for enhancing α-casein synthesis efficiency (9). Therefore, this study employed *in vitro* cultured MAC-T cells as an experimental model to investigate the effects of varying concentrations of valine, lysine, and threonine on α-casein synthesis and applied response surface methodology to determine their optimal combination. We further examined the impact of this optimal amino acid combination on α-casein synthesis, expression of encoding genes, and the transcriptional and post-translational regulation of mTOR signaling pathway components—including gene expression and protein phosphorylation. Additionally, we assessed these parameters following pharmacological inhibition of the mTOR pathway to evaluate whether the stimulatory effects of the amino acid combination depend on mTOR signaling. The study aims to elucidate the central role of the mTOR pathway in mediating the synergistic enhancement of α-casein synthesis by the three amino acids, thereby providing a scientific foundation for developing precise nutritional strategies in dairy cow management.

## 2 Methods

### 2.1 MAC-T cells

The bovine mammary epithelial cell line (MAC-T) used in this study was obtained from the cell bank of Qingqi (Shanghai) Biotechnology Co., Ltd. This cell line exhibits typical biological features of bovine mammary epithelial cells: following continuous *in vitro* passaging, the cells display stable adherence and form a confluent monolayer with a characteristic cobblestone-like mosaic pattern, featuring tight intercellular junctions and well-defined cellular boundaries. Over 30 passages, the population maintained greater than 95% morphological uniformity, with no signs of abnormal vacuolization or fibrotic transformation observed. These properties confirm that the MAC-T cell model reliably recapitulates the anabolic functions of lactating mammary epithelial cells, thereby fully satisfying the requirements for experimental system establishment in this study.

### 2.2 Materials

In this study, L-Val was purchased from Sigma (Cat No. V0513, sigma, St. Louis, Mo, USB), L-lysine was purchased from Sigma (Cat No. I7403-25G, sigma, St. Louis, Mo, USB), L-threonine was purchased from Sigma (Cat No. S4311-25G, sigma, St. Louis, Mo, USB). Dulbecco’s Modified Eagle Medium (DMEM, Cat No. C11965500BT, Thermo Scientific, Waltham, MA, United States), Fetal Bovine Serum (FBS, Cat No. 10099-141C, Thermo Scientific, Waltham, MA, United States) and 0.25% Trypsin–EDTA (Cat No. 25200-056, Thermo Scientific, Waltham, MA, United States) were purchased from Thermo Scientific. Rapamycin was purchased from Selleck (Cat No. S1039, Selleck Chemicals LLC, Houston, Texas, United States). PBS was purchased from Pricella (Cat No. Cat No. PB180327, Wuhan Pricella Biotechnology Co., Ltd., Wuhan, China).

### 2.3 Experimental design

Third-generation MAC-T cells were cultured for 24 h and then subjected to serum starvation for 12 h prior to treatment. The cells were subsequently randomized into 47 experimental groups, with three replicates per group. The positive control (PC) group was supplemented with 10% fetal bovine serum (FBS), while the negative control (0 × AA) group was maintained in serum-free medium. Experimental treatments included supplementation of valine (0 × AA, 0.25 × Val, 0.5 × Val, 1 × Val, 2 × Val, 4 × Val, and 8 × Val), lysine (0 × AA, 0.25 × Lys, 0.5 × Lys, 1 × Lys, 2 × Lys, 4 × Lys, and 8 × Lys), or threonine (0 × AA, 0.25 × Thr, 0.5 × Thr, 1 × Thr, 2 × Thr, 4 × Thr, and 8 × Thr) individually; various amino acid combinations as defined by response surface design; the optimal amino acid combination (MIX group); rapamycin alone; and rapamycin combined with the optimal amino acid mixture (Rapamycin + MIX). ELISA was used to quantify α-casein synthesis in MAC-T cells following individual supplementation with graded levels of valine, lysine, or threonine, as well as with different amino acid combinations. The optimal amino acid combination was identified using response surface methodology (RSM), and ELISA was further employed to compare α-casein synthesis under conditions of individual optimal amino acid concentrations versus the optimal combination. RT-qPCR was performed to assess the relative mRNA expression levels of the α-casein encoding gene and key genes in the mTOR signaling pathway. Western blot analysis was conducted to evaluate the effects of the optimal amino acid combination on phosphorylation of downstream mTOR pathway proteins and on α-casein synthesis. Integrated analysis of RT-qPCR and Western blot results was carried out to elucidate the stimulatory effect of the optimal amino acid combination on α-casein production and its potential activation of the mTOR signaling pathway. To validate the functional role of mTOR signaling, RT-qPCR and Western blot assays were specifically applied to four critical groups: 0 × AA, MIX, Rapamycin, and Rapamycin + MIX, to measure gene expression, protein phosphorylation levels, and α-casein synthesis. The concentrations of amino acids, serum, and rapamycin in each treatment group are detailed in Tables 1, 2. Due to the large number of combinations generated by the response surface design (*n* = 18), they are not listed individually in the text.

**Table 1:** Amino acid and serum levels in the culture medium of the single addition of valine, lysine and threonine test groups.

Items	DMEM medium (basic)	Groups^a^							
		0 × (DMEM only)	0.25×	0.5×	1×	2×	4×	8×	PC (10% FBS)
Valine (mmol/L)	0.803^b^	0.803	1.596	3.191	6.382	12.764	25.528	51.056	0.818^c^
Lysine (mmol/L)	0.798^b^	0.798	1.841	3.682	7.364	14.727	29.454	58.908	0.799^c^
Threonine (mmol/L)	0.798^b^	0.798	0.736	1.473	2.946	5.891	11.782	23.564	0.810^c^
FBS (%)	0.000	0.000	0.000	0.000	0.000	0.000	0.000	0.000	10.000

a The levels of valine, lysine and threonine set in this study were based on the optimal addition level of leucine determined by our team in previous research (when the synthesis of α-casein was the highest and the proliferation rate of MAC-T cells was the best), and were calculated based on the proportion of these three amino acids in the α-casein polypeptide chain. To ensure the rigor of the experimental design, after determining the 1 × basal level, three concentration gradients were added upwards and two concentration gradients were added downwards for each of the three amino acids.

b The concentrations of valine, lysine and threonine in the DMEM basal medium are 0.803 mmol/L, 0.798 mmol/L, and 0.798 mmol/L, respectively (calculated based on the formula provided by the manufacturer).

c The contribution of FBS was estimated based on the typical concentrations of free amino acids in bovine serum as reported by Ghaffari et al. (38). Although there are fluctuations at physiological levels (valine: 0.10–0.15 mmol/L, lysine: 0.08–0.10 mmol/L, threonine: 0.08–0.12 mmol/L), the maximum estimated values (valine: 0.15 mmol/L, lysine: 0.10 mmol/L, threonine: 0.12 mmol/L) were adopted as the concentrations of each amino acid in FBS, considering that the concentration of free amino acids in fetal bovine serum may be higher than that in adult bovine plasma. Therefore, the contributions of valine, lysine and threonine in 10% FBS are 0.015, 0.01 and 0.012 mmol/L, respectively.

**Table 2:** Amino acid, serum, and rapamycin concentrations in the culture medium of experimental groups under mTOR signaling pathway inhibition.

Items	Groups			
	0 × AA	MIX	Rapamycin	Rapamycin + MIX
Valine (mmol/L)	0.000	36.113	0.000	36.113
Lysine (mmol/L)	0.000	9.025	0.000	9.025
Threonine (mmol/L)	0.000	4.602	0.000	4.602
Rapamycin (nM)	0.000	0.000	100.000^a^	100
FBS (%)	0.000	0.000	0.000	0.000

a The concentration of rapamycin used (100 nM) was determined based on the previous study by Yang et al. (1) of our team. This concentration can effectively inhibit the activity of the mTOR signaling pathway in MAC-T cells.

### 2.4 Detection parameters and analytical methods

#### 2.4.1 Response surface methodology experimental design

To determine the optimal combination of valine, lysine, and threonine for enhancing α-casein production, a three-factor, five-level central composite design (CCD) was implemented using response surface methodology software (Design-Expert 13). The concentrations of valine, lysine, and threonine were designated as independent variables (coded as A, B, and C), with five levels for each factor determined based on the effective concentration ranges identified in preliminary single-factor experiments (Val: 6.382–25.528 mmol/L; Lys: 1.841–14.727 mmol/L; Thr: 0.736–5.891 mmol/L). The coded levels −2, −1, 0, +1, and +2 corresponded to the actual low, mid-low, central, mid-high, and high concentration levels of each amino acid, respectively (experimental factors and their levels are presented in Table 3). A total of 18 experimental combinations of these three amino acids were generated according to the CCD matrix and are listed in Table 4.

**Table 3:** Coding values of experimental factors for the response surface methodology optimization of valine, lysine, and threonine in enhancing α-casein expression.

Factors	Levels				
	−2	−1	0	+1	+2
A-Val (mmol/L)	6.382	17.5505	28.719	39.8875	51.056
B-Lys (mmol/L)	1.841	5.0625	8.248	11.5055	14.727
C-Thr (mmol/L)	0.736	2.02475	3.3135	4.60225	5.891

**Table 4:** Response surface methodology experimental design for optimizing α-casein expression through valine, lysine, and threonine.

Test number	A (Val)	B (Lys)	C (Thr)	Y_1_ (α-casein)
1	−1	−1	−1	
2	1	-1	−1	
3	−1	1	−1	
4	1	1	−1	
5	−1	−1	1	
6	1	−1	1	
7	−1	1	1	
8	1	1	1	
9	−2	0	0	
10	2	0	0	
11	0	−2	0	
12	0	2	0	
13	0	0	−2	
14	0	0	2	
15	0	0	0	
16	0	0	0	
17	0	0	0	
18	0	0	0	

A denotes the coded value of Val; B denotes the coded value of Lys; C denotes the coded value of Thr.

#### 2.4.2 Cell culture and processing

All the detection indicators in this study were performed using the third-generation MAC-T cells. The cells were harvested by conventional trypsin digestion and then collected by centrifugation at 1000 rpm for 10 min using a low-speed benchtop centrifuge (Model: LT53, Hunan Xiangyi Laboratory Instrument Equipment Co., Ltd., Hunan, China). The cells were then resuspended in DMEM containing 10% fetal bovine serum (FBS), and the cell density was determined using a Countess 3 FL Automated Cell Counter (Model: AMQAF2000, Thermo Fisher Scientific, Waltham, MA, United States). The cells were seeded into the corresponding culture plates or flasks at the required density for each indicator and cultured in a 37 °C, 5% CO2 incubator (Model: WCI-180, VIGON GmbH, Baden-Württemberg, Germany) for 24 h to allow the cells to adhere. Subsequently, the medium was replaced with serum-free DMEM and the cells were starved for 12 h to synchronize the cell cycle. Thereafter, different treatment media were added according to the experimental design, and the cells were collected at the 12-h time point determined by the MTT method for subsequent detection.

#### 2.4.3 Determination of the relative proliferation rate of cells

Cell proliferation was assessed using the MTT assay and expressed as the relative growth rate (RGR). After culturing and synchronizing the cells according to the method described in “2.4.2,” the cells were seeded into 96-well plates (Catalog No.: CLS351172, Corning Inc., Corning, NY, United States) at a density of 5 × 10^3^ cells per well, with 100 μL of medium containing 10% FBS in each well. Two hundred microliter of PBS was added to the peripheral wells of the plate to reduce edge effects. After 24 h of adherent culture, the cells were starved for 12 h and then 100 μL of treatment medium containing different combinations of valine, lysine and threonine was added to each well. At each time point of 2, 4, 6, 12, 24, and 48 h of culture, the medium in the corresponding wells was discarded and 50 μL of MTT solution (0.5 mg/mL, Catalog No.: M5655-500MG, Sigma-Aldrich, St. Louis, MO, United States) was added to each well and incubated for 4 h. The supernatant was discarded and 150 μL of DMSO (Catalog No.: D4540-100 mL, Sigma-Aldrich, St. Louis, MO, United States) was added to each well and shaken in the dark for 8–10 min to dissolve the formazan crystals. The absorbance value at 490 nm was measured using a microplate reader (Infinite 200Pro, Tecan GmbH, Männedorf, Switzerland).

$\begin{array}{l} \text{Calculation formula}:\mathrm{RGR}\;(\%)\\ =(\begin{array}{l} {\mathrm{OD}}_{490}\;\text{of the test group}\\ /{\mathrm{OD}}_{490}\;\text{of the control group} \end{array})\times 100\%. \end{array}$

#### 2.4.4 Determination of α-casein

The synthesis of α-casein in MAC-T cells was determined using an enzyme-linked immunosorbent assay (ELISA). After culturing and synchronizing the cells according to the method described in “2.4.2,” the cells were seeded at a density of 6 × 10^6^ cells per T25 flask in 6 mL of medium containing 10% FBS. After 24 h of culture for adherence and starvation treatment, the medium was discarded and 6 mL of treatment medium with different levels of valine, lysine, threonine or their different combinations was added to each flask. At the 12-h sample collection time point, cells were collected and lysed by ultrasonication for 5 min at 80 kHz using a cell disruptor (Cat No. FB50, Thermo Scientific, Waltham, MA, United States) to release intracellular proteins. The operation was carried out according to the instructions of the bovine α-casein ELISA kit (Cat No. JL22469, Shanghai Jianglai Biotechnology Co., Ltd., Shanghai, China): The standard substances and the samples to be tested (3 replicates for each group) were added to the corresponding wells of the microplate. Except for the blank well, 100 μL of HRP-labeled antibody was added to each well. The plate was sealed and incubated in a water bath at 37 °C in the dark for 1 h. The liquid was discarded, and the plate was washed 5 times with 350 μL of washing solution, with an interval of 3 min each time. The plate was patted dry. Fifty microliter of substrate solution (A solution and B solution were mixed in equal volume) was added to each well, and the plate was incubated at 37 °C in the dark for 15 min. Fifty microliter of stop solution was added to each well to terminate the reaction, and the absorbance value at 450 nm was measured within 15 min using a microplate reader. The intracellular α-casein concentration was calculated based on the standard curve.

#### 2.4.5 Determination of the expression levels of related genes

Total RNA was extracted from MAC-T cells using Trizol reagent (Cat No. 15596026CN, Thermo Fisher Scientific, Waltham, MA, United States) according to the manufacturer’s instructions. RNA purity and concentration were assessed spectrophotometrically for each sample prior to reverse transcription. High-quality total RNA was then reverse transcribed into cDNA using a reverse transcription kit (Cat No. RR420A, TaKaRa Bio Inc., Shiga, Japan). The relative mRNA expression levels of the α-casein-encoding gene (*CSN1S1*) and key regulatory genes in the mTOR signaling pathway were quantified by real-time fluorescent quantitative PCR (RT-qPCR). Amplification was performed using the same RT-qPCR system (TaKaRa), and β-actin (ACTB) was used as the internal reference gene to normalize gene expression across samples. The relative expression levels of target genes were calculated using the 2^−ΔΔCt^ method. Primer sequences for all genes are provided in Table 5. The formula for calculating the relative expression level of the target gene is as follows:

**Table 5:** Primer sequences of internal reference genes and target genes.

Genes	Gene accession number	Primer sequence
*mTOR*	XM_001788228.1	F: ATGCTGTCCCTGGTCCTTATG
		R: GGGTCAGAGAGTGGCCTTCAA
*MLST8*	NM_001035411	F: ATCTGTACGCGAACTGTGCAG
		R: CATACATGCGAATGTGCTGG
*Raptor*	NM_001192130	F: GTTTGGAATGCTGGATTGATC
		R: CTGAGTGTAGTTCTTGTTGAAGACC
*RRAGA*	NM_001035499	F: GAGGTTCTGATTTATGTGTTCGAC
		R: ACAATACTGGACCAGGCTTTGTAG
*RRAGB*	NM_001075279	F: GGGTAAGACCAGCATGAGGTCT
		R: CACAGTCCCACAGGTTCAATACC
*RRAGC*	NM_001076456	F: CATCCAGAAGGTGGTGTTTCAT
		R: GCATCAATGACATATATCAATGCTC
*RRAGD*	NM_001192828	F: CAGAGGTAAAGCCGAGGATCC
		R: TCCAAGAACAGAGTTTCGTTGG
*S6K1*	NM_205816.1	F: CTGGGGAAGAGGTGCTTCAG
		R: GTGCTCTGGTCGTTTGGAGA
*EIF4EBP1*	BC120290.1	F: GAACTCACCTGTGACCAAGA
		R: CTCAAACTGTGACTCTTCACC
*EIF4E*	NM_174310.1	F: ACGAAGTGACCTCGATCGTT
		R: AGTAGCTGTGTCTGCATGGG
*EEF2*	NW_618521.1	F: CTCTACCAAACCTTCCAGCG
		R: GCTGTTGGCTGACTTGCTGA
*Rps6*	NM_001010.2	F: AAGAGCTAGCAGAATCCGCA
		R: CGTGGAGTAACAAGACGCTG
*CSN1S1*	NM_181029.2	F: TCAACCCAGCTTGCTGCTTCTTCC
		R: GCCTAGCAAGAGCAACAGCCACAA
*CSN1S2*	NM_174528.2	F: AGCAGCTCTCCACCAGTGAGGAAA
		R: TGGGGCAAGGCGAATTTCTGGT
*β-actin*	NM_173979.3	F: GTCATCACCATCGGCAATGAG
		R: AATGCCGCAGGATTCCATG

$\mathrm{\Delta Ct}={\mathrm{Ct}}_{\text{target}}–{\mathrm{Ct}}_{\text{reference gene}}$

$\Delta \Delta \mathrm{Ct}=\mathrm{\Delta C}t_{\text{treatment}}–\mathrm{\Delta C}t_{\text{control}\;(0\times \mathrm{AA})}$

$\text{Relative expression}=2^{-\Delta \Delta \mathrm{Ct}}$

#### 2.4.6 Determination of phosphorylation of related proteins

The cell culture protocol for the Western blot analysis was identical to that used in the RT-qPCR experiment of this study. After treating MAC-T cells with amino acids, rapamycin, and 10% FBS for 12 h, the medium in the 6-well plates was discarded. Each well was washed 2–3 times with pre-cooled PBS to remove residual medium. Subsequently, 100 μL of RIPA lysis buffer (Cat No. AS1004, Pharmacare Holdings Limited, Johannesburg, South Africa), supplemented with 1% phosphatase inhibitor (Cat No. AS1008, Pharmacare Holdings Limited, Johannesburg, South Africa) and 1% protease inhibitor (Cat No. 04693159001, Roche Holding AG, Basel, Switzerland), was added to each well and incubated for 3–5 min to lyse the cells. The lysates were collected into 1.5 mL centrifuge tubes using a cell scraper. After a 30 min incubation on ice, the samples were centrifuged at 12,000 rpm for 5 min at 4 °C in a low-temperature high-speed centrifuge, and the supernatants, representing the total protein solution, were collected. The protein concentration of each samplhe from the difhferent treatment groups was determined using a BCA protein quantification kit (Cat No. AS1086, Pharmacare Holdings Limited, Johannesburg, South Africa), and the samples were adjusted to a uniform concentration. Equal amounts (40 μg) of protein were mixed with 5 × protein loading buffer at a volume ratio of 4:1, boiled at 100 °C for 5 min, and prepared for electrophoresis. The proteins were subjected to SDS-PAGE electrophoresis and then transferred to a PVDF membrane (Cat No. IPVH00010, Millipore Corporation, Bedford, MA, United States). The membrane was blocked with blocking buffer (Cat No. P0023B, Shanghai Beyotime Biotechnology Co., Ltd., Shanghai, China) at room temperature for 1 h. After removing the blocking buffer, diluted primary antibodies were added, and the membrane was incubated overnight at 4 °C. The next day, the PVDF membrane was washed three times with TBST [prepared by mixing 1,000 mL of TBS solution (Cat No. AS1024, Pharmacare Holdings Limited, Johannesburg, South Africa) with 1 mL of Tween-20 solution (Cat No. AS1100, Pharmacare Holdings Limited, Johannesburg, South Africa)] for 5 min each time. Secondary antibodies were then added, and the membrane was incubated at room temperature for 30 min, followed by four washes with TBST on a shaking platform at room temperature. Finally, the ECL chemiluminescent detection kit (Cat No. AS1059, Pharmacare Holdings Limited, Johannesburg, South Africa) was used to prepare a fresh ECL mixture (A:B = 1:1), which was applied to the protein side of the membrane. The membrane was exposed and developed in a darkroom. The films were scanned and archived, and the optical density values of the target bands were analyzed using the AlphaEaseFC software version 4.0 processing system. Detailed information about these antibodies is presented in Table 6.

**Table 6:** Detailed information of antibodies.

Antibody Type	Antibody Name	Phosphorylation sites	Source	Catalog number	Dilution method	Dilution ratio
Primary antibody	β-actin		Tian De Yue	TDY051	5% fat-free milk	1:10000
	P-mTOR	Ser2448	bioss	bs-3494R	5% egg white	1:500
	P-S6K1	Thr389/Thr412	affbiotech	AF3228	5% egg white	1:1000
	S6K1		affbiotech	AF6226	5% egg white	1:2000
	P-4EBP1	Thr45	affbiotech	AF3432	5% egg white	1:1000
	4EBP1		affbiotech	AF6432	5% egg white	1:2000
	P-RPS6	Ser235	affbiotech	AF3354	5% egg white	1:500
	RPS6		biorbyt	orb585017	5% egg white	1:1000
	P-eFF2	Tyr443	affbiotech	AF7220	5% egg white	1:500
	eEF2		biorbyt	orb584002	5% egg white	1:1000
Secondary antibody	HRP-goat anti rabbit		ASPEN	AS1107	5% fat-free milk	1:10000
	HRP-goat anti mouse		ASPEN	AS1106	5% fat-free milk	1:10000
	HRP-rabbit anti goat		ASPEN	AS1108	5% fat-free milk	1:10000
	HRP-goat anti rat		ASPEN	AS1093	5% fat-free milk	1:10000
	HRP-rabbit anti sheep		ASPEN	AS1245	5% fat-free milk	1:10000

### 2.5 Statistical analysis

All data were calculated and organized using Excel 2016 and analyzed statistically with SPSS 19.0. For comparisons among multiple groups, one-way analysis of variance (ANOVA) was used. When significant differences were found among groups, the Duncan’s new multiple range test was applied for multiple comparisons. For comparisons between two groups, the independent samples *t*-test was used to analyze the significance of differences. *p* < 0.05 indicated a significant difference, and *p* < 0.01 indicated a highly significant difference.

## 3 Results

### 3.1 Effects of individually supplementing varying concentrations of valine, lysine, or threonine on α-casein synthesis in MAC-T cells

**Figure 1:**
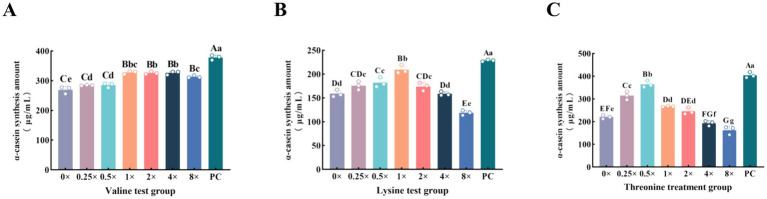
Effects of individual supplementation with three concentration levels of amino acids on intracellular α-casein synthesis. **(A–C)** Respectively represent the amounts of α-casein synthesized by MAC-T cells after being cultured for 12 h with the supplementation of different concentrations of valine, lysine, and threonine. The error bars represent the SD (*n* = 3). Data were analyzed by one-way ANOVA, and the *post hoc* multiple comparisons were conducted using Duncan’s new multiple range test. Different lowercase letters in the figures indicate significant differences (*p* < 0.05), and different uppercase letters indicate extremely significant differences (*p* < 0.01).

To enable a direct comparison with α-casein synthesis levels achieved under optimal combinatorial amino acid supplementation, we employed ELISA to determine the individually optimal supplementation levels of valine, lysine, and threonine (Figure 1). The results indicated that α-casein synthesis in MAC-T cells exhibited a biphasic response to increasing supplementation levels of valine, lysine, and threonine, initially increasing and subsequently decreasing at higher concentrations. Compared with the 0 × Val group, supplementation with valine at 0.25 × Val and 0.5 × Val significantly increased intracellular α-casein synthesis (*p* < 0.05). Supplementation with valine at 1 × Val, 2 × Val, 4 × Val, and 8 × Val significantly increased intracellular α-casein synthesis compared to the control (*p* < 0.01), with peak levels observed at the 4 × Val group, where α-casein synthesis was 4.268 and 0.152% higher than at 1 × Val and 2 × Val, respectively (Figure 1A). Supplementation with lysine at 0.5 × Lys and 1 × Lys significantly increased intracellular α-casein synthesis (*p* < 0.01), while supplementation at 0.25 × Lys and 2 × Lys also enhanced synthesis (*p* < 0.05). However, higher concentrations at 4 × Lys and 8 × Lys markedly suppressed α-casein synthesis (*p* < 0.01), with peak levels observed at the 1 × Lys group (Figure 1B). Supplementation with threonine at 0.25 × Thr, 0.5 × Thr, and 1 × Thr significantly increased intracellular α-casein synthesis (*p* < 0.01), while supplementation at 2 × Thr also enhanced synthesis (*p* < 0.05). In contrast, α-casein synthesis was significantly reduced at 4 × Thr (*p* < 0.05) and markedly suppressed at 8 × Thr (*p* < 0.01). The highest level of α-casein synthesis was observed at the 0.5 × Thr group (Figure 1C). These results indicate that the optimal addition ranges for enhancing α-casein synthesis are 1 × Val–8 × Val for valine, 0.25 × Lys–2 × Lys for lysine, and 0.25 × Thr–2 × Thr for threonine, with the respective optimal concentrations being 4 × Val, 1 × Lys, and 0.5 × Thr.

### 3.2 Effects of various combinations of valine, lysine, and threonine on the relative proliferation rate of MAC-T cells

**Figure 2:**
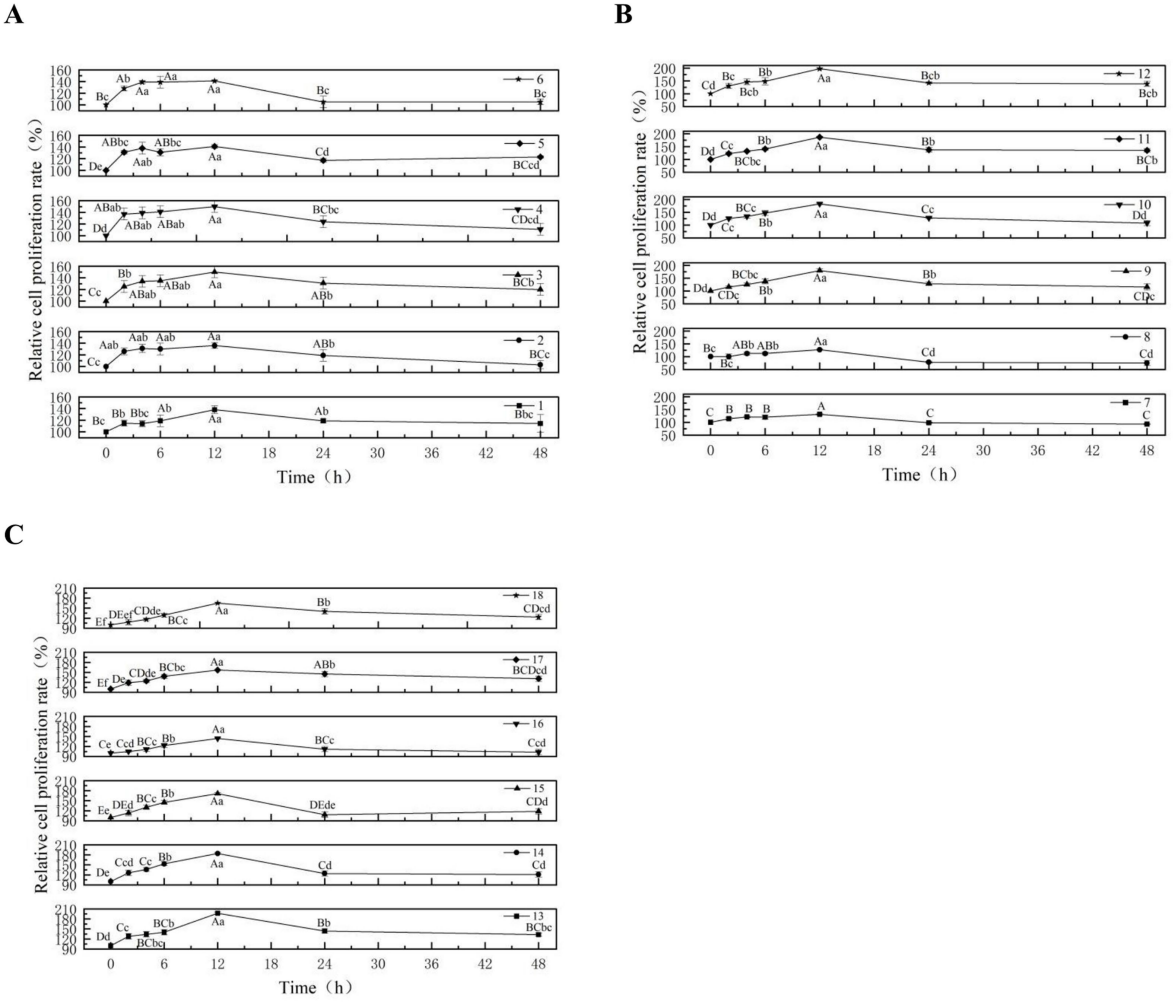
Effects of different levels of valine, lysine and threonine on the relative proliferation rate of MAC-T cells. **(A)** Illustrates the effects of varying concentration ratios of valine, lysine, and threonine on the relative proliferation rate of MAC-T cells in groups 1–6, **(B)** depicts the corresponding effects in groups 7–12, and **(C)** presents the results for groups 13–18. After culturing the MAC-T cells in groups 1–18 under different amino acid combinations for 0, 2, 4, 6, 12, 24, and 48 h respectively, the proliferation rates of the cells were detected. The error bars represent the SD (*n* = 3). Data were analyzed by one-way ANOVA, and the *post hoc* multiple comparisons were conducted using Duncan’s new multiple range test. Different lowercase letters in the figures indicate significant differences (*p* < 0.05), and different uppercase letters indicate extremely significant differences (*p* < 0.01).

To identify the optimal culture duration among 18 distinct combinations of valine, lysine, and threonine ratios, we systematically measured the cell growth rate for each combination and thereby determined the most favorable culture time for the amino acid formulations (The numbers 1–18 in the figure, respectively, represent the 1–18 groups of different combinations of valine, lysine and threonine test groups.). The results demonstrated that upon supplementation with various ratios of valine, lysine, and threonine, the relative proliferation rate of MAC-T cells initially increased and subsequently decreased as culture time was prolonged (Figures 2A–C). Compared to the 0 h baseline, groups 1, 2, 3, 4, 11, 12, 13, 14, 15, 17, and 18 showed extremely significant increases in cell proliferation throughout the 2–48 h culture period (*p* < 0.01). In groups 1–4, although the proliferation rates at 12 h were not significantly different from those at 6 h within the same group (*p* > 0.05), they were 15.97, 4.62, 11.11, and 6.38% higher, respectively. For groups 11, 12, 13, 14, 15, 17, and 18, the relative proliferation rate peaked at 12 h, showing extremely significant increases compared to 0 h and all other time points within each group (*p* < 0.01). In group 5, the proliferation rate at 12 h was significantly higher than at 2 h and 6 h (*p* < 0.05), extremely significantly higher than at 0 h and 48 h (*p* < 0.01), and 2.17% higher than at 4 h. In group 6, the rate at 12 h was extremely significantly elevated compared to 0 h, 24 h, and 48 h (*p* < 0.01), and was 10.16, 1.44, and 1.44% higher than at 2 h, 4 h, and 6 h, respectively. Groups 7, 9, 10 and 16 also exhibited peak proliferation at 12 h, with extremely significant differences compared to all other time points (*p* < 0.01). In group 8, the proliferation rate at 12 h was extremely significantly higher than at 0 h, 2 h, 24 h and 48 h (*p* < 0.01), and significantly higher than at 4 h and 6 h (*p* < 0.05). Collectively, across all 18 amino acid combination groups tested, the relative proliferation rate of MAC-T cells reached its maximum at 12 h. Therefore, 12 h was selected as the optimal sampling time for subsequent experiments.

### 3.3 The optimal combination of valine, lysine and threonine for the synthesis of α-casein was screened by response surface design

To investigate the effect of the optimal combination of valine, lysine, and threonine on α-casein synthesis in MAC-T cells, an *in vitro* cell culture model was employed. Following a 12-h serum starvation period, cells were exposed to culture media supplemented with varying ratios of these amino acids. As shown in Tables 7, 8, α-casein synthesis reached its maximum when valine, lysine, and threonine were added at concentrations of 36.114 mmol/L, 9.027 mmol/L, and 4.602 mmol/L, respectively—corresponding to a molar ratio of 8:2:1.

**Table 7:** Response surface methodology for optimizing α-casein expression: experimental design and results using valine, lysine, and threonine.

Test number	A (Val)	B (Lys)	C (Thr)	Y_1_ (α-casein)
1	−1	−1	−1	378.455
2	1	−1	−1	330.707
3	−1	1	−1	338.744
4	1	1	−1	310.78
5	−1	−1	1	359.073
6	1	−1	1	365.171
7	−1	1	1	349.024
8	1	1	1	380.915
9	−2	0	0	354.585
10	2	0	0	339.691
11	0	−2	0	338.841
12	0	2	0	318.715
13	0	0	−2	320.085
14	0	0	2	369.256
15	0	0	0	391.732
16	0	0	0	389.341
17	0	0	0	368.61
18	0	0	0	376.63

A denotes the coded value of Val. B denotes the coded value of Lys. C denotes the coded value of Thr. Y1 (α-casein) represents the response value, indicating the amount of α-casein synthesized in MAC-T cells (unit: ng/mL).

**Table 8:** Screening of optimal amino acid combinations of valine, lysine, and threonine for α-casein synthesis: results from response surface methodology optimization.

Sequence	Val	Lys	Thr	α-casein	Desirability	Selection status
1	36.114	9.027	4.602	388.393	0.883	Selected
2	36.039	9.009	4.602	388.392	0.883	
3	36.199	9.039	4.602	388.392	0.883	
4	36.159	9.004	4.602	388.392	0.883	
5	36.150	8.978	4.602	388.389	0.883	
6	36.318	9.074	4.602	388.388	0.883	
7	35.847	9.014	4.602	388.388	0.883	
8	35.965	9.078	4.602	388.387	0.883	
9	35.760	9.046	4.602	388.382	0.883	
10	36.446	8.992	4.602	388.382	0.883	
11	36.301	8.965	4.602	388.381	0.883	
12	35.532	8.953	4.602	388.369	0.883	
13	35.580	8.916	4.602	388.367	0.883	
14	36.305	9.181	4.602	388.364	0.883	
15	36.305	8.806	4.602	388.322	0.882	
16	35.309	9.228	4.602	388.271	0.882	
17	35.437	8.560	4.602	388.136	0.880	
18	33.856	9.041	4.602	388.038	0.879	

As shown in Tables 9, 10, using *in vitro* cultured MAC-T cells as a model, different concentrations and combinations of valine, lysine, and threonine were supplemented into the culture medium. The response surface regression analysis revealed that these three essential amino acids (EAAs) had a significant effect on α-casein expression (*p* < 0.01, *R*^2^ = 0.9640), while the lack-of-fit was not significant (*p* = 0.9977 > 0.05), indicating that the model fits the experimental data well and is statistically valid. The overall *F*-value of the regression model was 23.80, and based on the individual *F*-values, the relative influence of each factor on α-casein expression followed the order: threonine (*F* = 49.24, *p* = 0.0001) > lysine (*F* = 11.63, *p* = 0.0092) > valine (*F* = 5.97, *p* = 0.0403). To determine the optimal combination of these EAAs, a quadratic regression analysis was performed to establish the relationship between the independent variables and the response variable. A second-order polynomial equation was applied for modeling. Using Design-Expert 13 software, multiple regression fitting was conducted on the data from Table 9, with valine, lysine, and threonine coded as factors A, B, and C, respectively, and α-casein expression set as the response. The resulting quadratic multiple regression equation was obtained as follows:

**Table 9:** Regression analysis results for the α-casein production model and estimated regression coefficients.

Source	Sum of squares	df	Mean square	*F*-value	*P*-value
Model	10216.75	9	1135.19	23.80	<0.0001
A-Val	284.86	1	284.86	5.97	0.0403
B-Lys	554.54	1	554.54	11.63	0.0092
C-Thr	2348.35	1	2348.35	49.24	0.0001
AB	259.66	1	259.66	5.44	0.0479
AC	1615.99	1	1615.99	33.88	0.0004
BC	533.55	1	533.55	11.19	0.0102
A^2^	1590.83	1	1590.83	33.35	0.0004
B^2^	3760.76	1	3760.76	78.85	<0.0001
C^2^	1828.98	1	1828.98	38.35	0.0003
Residual	381.55	8	47.69		
Lack of fit	25.53	5	5.11	0.0430	0.9977
Pure error	356.02	3	118.67		
Cor total	10598.30	17			

**Table 10:** Correlation analysis results for the response surface model.

Item	Value	Item	Value
Std. Dev.	6.91	R-Squared	0.9640
Mean	304.46	Adj R-Squared	0.9235
C. V. %	1.95	Pred R-Squared	0.9234
		Adeq Precision	13.5459

$\begin{array}{l} Y=381.86-4.22^{*}A-5.89^{*}B+12.11^{*}C+5.70^{*}\mathrm{AB}\\ +14.21^{*}\mathrm{AC}+8.17^{*}\mathrm{BC}-8.54^{*}A^{2}-13.13^{*}B^{2}-9.16^{*}C^{2} \end{array}$

The multiple regression fitting was conducted with α-casein as the response value. The regression model coefficients and the results of significance tests are presented in Table 9.

**Figure 3:**
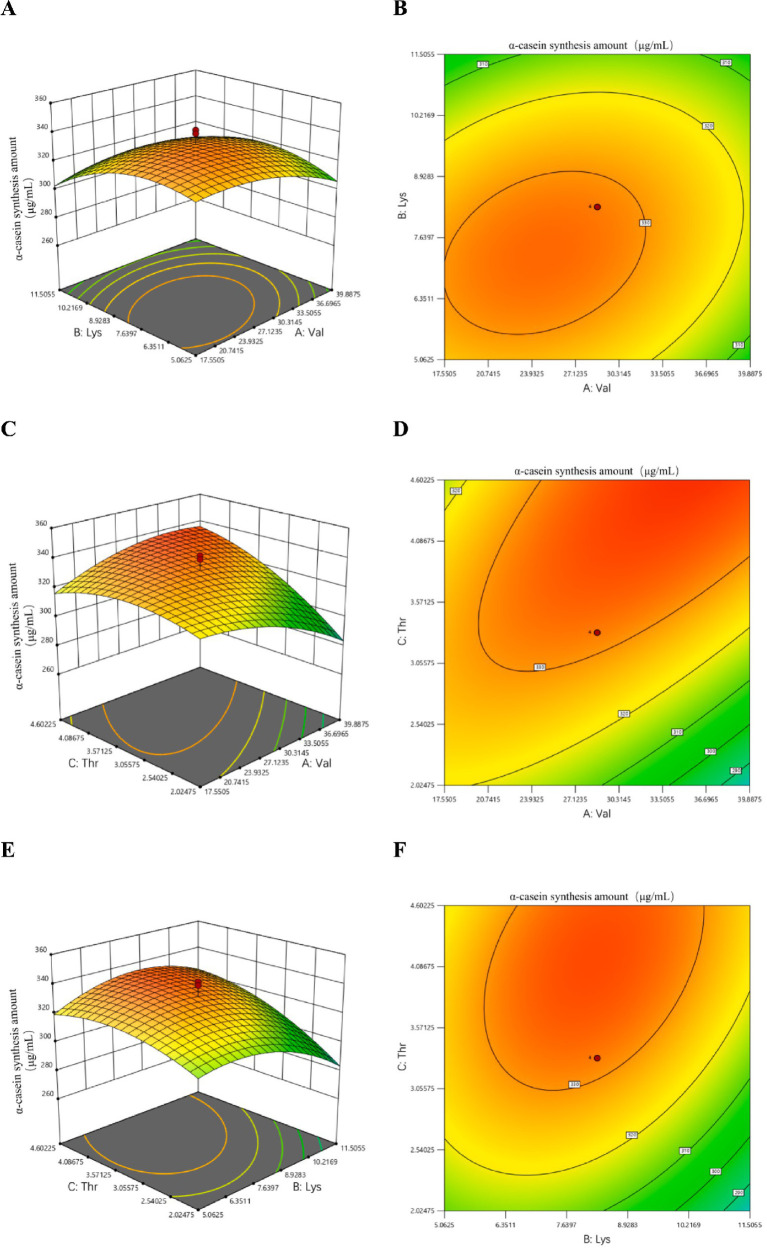
Response surface and contour plots illustrating the interactive effects of valine **(A)**, lysine **(B)**, and threonine **(C)** on α-casein synthesis. **(A,B)** Represent the response surface and contour plots illustrating the interaction between valine and lysine on α-casein synthesis. **(C,D)** Illustrate the interaction between valine and threonine on α-casein synthesis; and **(E,F)** depict the interaction between lysine and threonine on α-casein synthesis.

Based on the regression equation, the response surface plots and corresponding contour plots were analyzed to evaluate the effects of valine (A), lysine (B), and threonine (C) on α-casein synthesis. The interaction effects among valine (A), lysine (B), and threonine (C) on α-casein synthesis are presented in Figure 3.

As shown in Figures 3A,B, the interaction between lysine and valine exerts a significant influence on α-casein synthesis. In the response surface plot, when lysine levels are low, α-casein synthesis rapidly increases to a peak and then gradually declines with increasing valine concentration; in contrast, under high lysine conditions, synthesis initially rises slowly and subsequently decreases sharply as valine levels increase. This demonstrates that the effect of valine on α-casein synthesis varies depending on lysine levels, yet an optimal valine concentration exists at each level that maximizes synthesis. Overall, higher α-casein synthesis is achieved under high lysine conditions compared to low lysine conditions. Thus, rational modulation of the lysine-to-valine ratio can effectively enhance α-casein production. When considering only this pairwise interaction, maximum synthesis is likely attained when lysine concentrations range from 5.0625 to 11.5505 and valine concentrations range from 23.9325 to 39.8875.

As shown in Figures 3C,D, the interaction between threonine and valine exerts a significant influence on α-casein synthesis. In the response surface plot, when threonine levels are low, α-casein synthesis rapidly increases to a peak and then gradually declines with increasing valine concentration, in contrast, under high threonine conditions, synthesis initially rises slowly and subsequently decreases sharply as valine concentration increases. This demonstrates that the effect of valine on α-casein synthesis varies depending on threonine levels, yet an optimal valine concentration exists at each level that maximizes synthesis. Overall, α-casein synthesis is higher under high threonine conditions compared to low threonine conditions. Thus, rational modulation of the threonine-to-valine ratio can effectively enhance α-casein production. When considering only this pairwise interaction, maximum synthesis is likely attained when threonine concentrations range from 2.02475 to 3.57125 and valine concentrations range from 23.9325 to 39.8875.

As shown in Figures 3E,F, the interaction between threonine and lysine exerts a significant influence on α-casein synthesis. In the response surface plot, when threonine levels are low, α-casein synthesis rapidly increases to a peak and then gradually declines with increasing lysine concentration; in contrast, under high threonine conditions, synthesis initially rises slowly and subsequently decreases sharply as lysine concentration increases. This demonstrates that the effect of lysine on α-casein synthesis varies depending on threonine levels, yet an optimal lysine concentration exists at each level that maximizes synthesis. Overall, α-casein synthesis is higher under high threonine conditions compared to low threonine conditions. Thus, rational modulation of the threonine-to-lysine ratio can effectively enhance α-casein production. When considering only this pairwise interaction, maximum synthesis is likely attained when threonine concentrations range from 3.05575 to 4.08675 and lysine concentrations range from 6.3511 to 10.2169.

### 3.4 Effects of the optimal levels and combinations of valine, lysine, and threonine supplementation on α-casein synthesis

**Figure 4:**
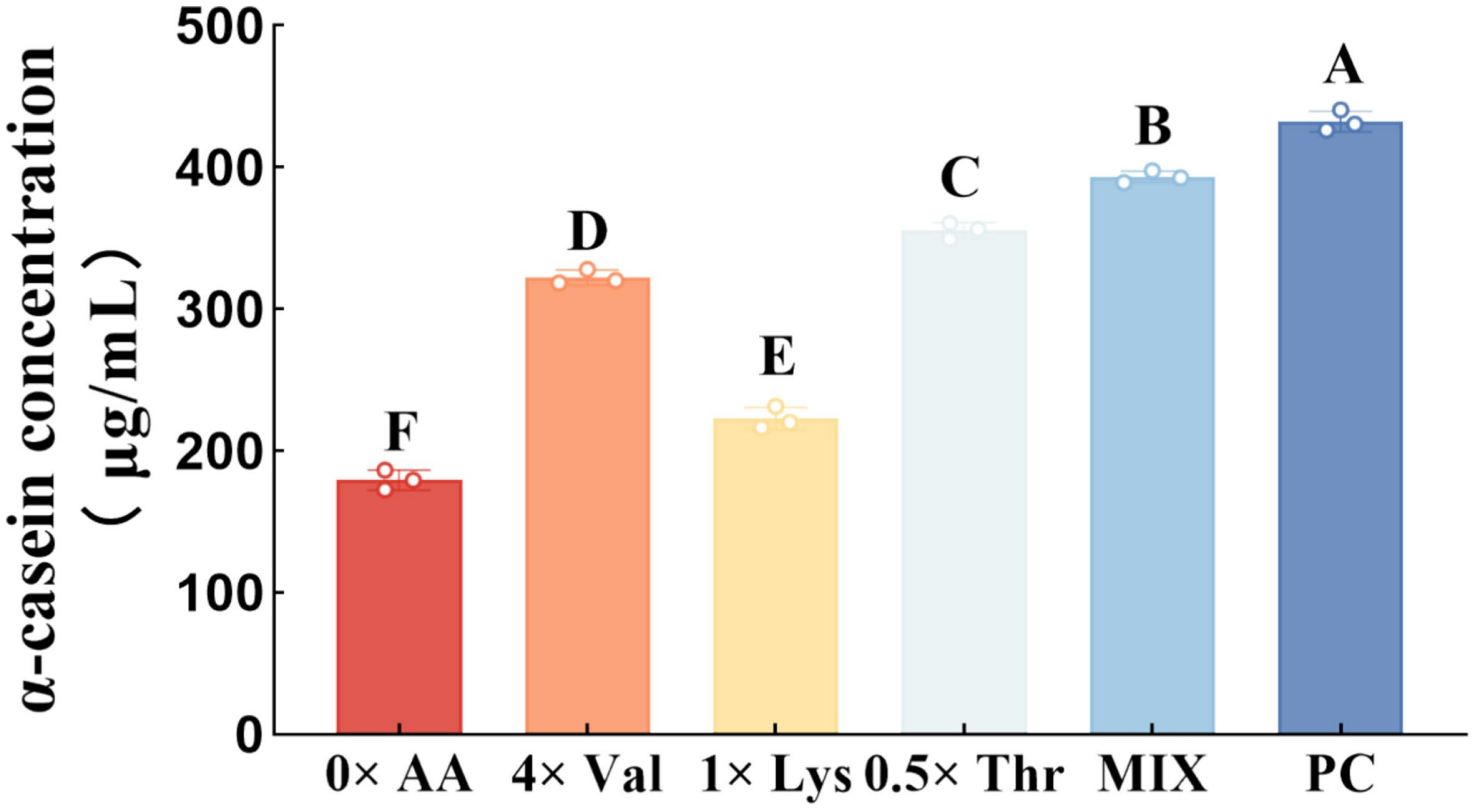
Effects of the optimal levels and ratios of valine, lysine, and threonine on α-casein synthesis (the detection was carried out 12 h after the amino acid supplementation treatment). The error bars represent the SD (*n* = 3). Data were analyzed by one-way ANOVA, and the post hoc multiple comparisons were conducted using Duncan’s new multiple range test. Different lowercase letters in the figures indicate significant differences (*p* < 0.05), and different uppercase letters indicate extremely significant differences (*p* < 0.01).

To validate whether the amino acid combination identified through response surface methodology could achieve maximal α-casein synthesis efficiency, this study compared the α-casein yield obtained with the optimized combination against the synthesis levels achieved under the individually optimized concentrations of valine, lysine, and threonine—previously determined via single-factor experiments—under identical experimental conditions (Figure 4). The results demonstrated that, compared with the 0 × AA group, supplementation with 4 × Val, 1 × Lys, 0.5 × Thr, or the MIX formulation significantly enhanced intracellular α-casein synthesis in MAC-T cells (*p* < 0.01). Notably, the MIX group exhibited significantly higher α-casein levels than the individual 4 × Val, 1 × Lys, and 0.5 × Thr groups (*p* < 0.01). In contrast, when compared with the PC group, all supplemented media—4 × Val, 1 × Lys, 0.5 × Thr, and MIX—significantly reduced intracellular α-casein synthesis (*p* < 0.01).

### 3.5 Effect of the optimal ratio of valine, lysine, and threonine supplementation on the expression of the α-casein coding gene

**Figure 5:**
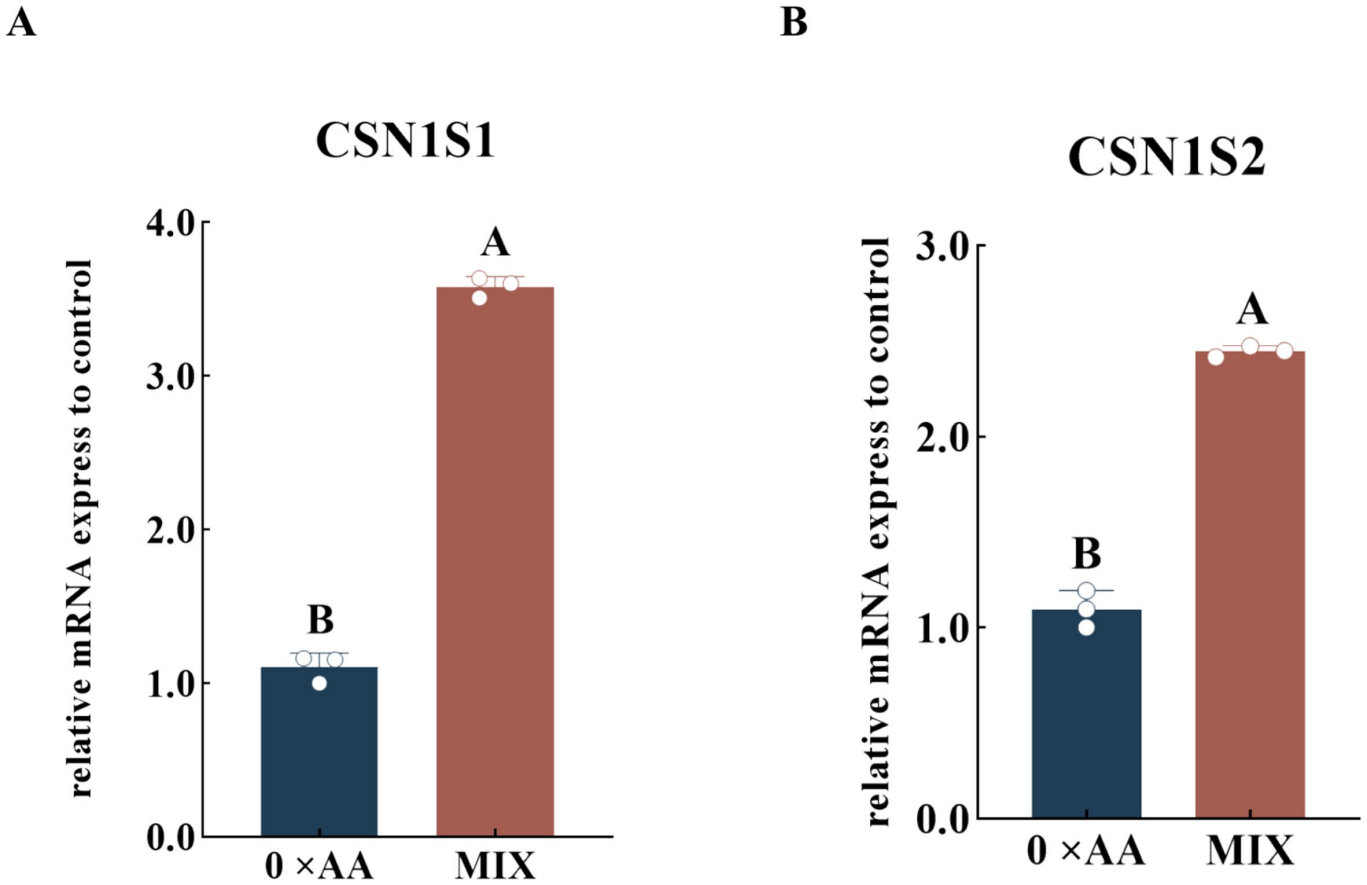
Effects of the optimal ratio of valine, lysine, and threonine on the relative expression level of the α-casein encoding gene (the detection was carried out 12 h after the amino acid supplementation treatment). **(A,B)** Represent the relative expression levels of the *CSN1S1* and *CSN1S2* genes, respectively. The error bars represent the SD (*n* = 3). The relative expression levels of genes were calculated using the 2^−ΔΔCt^ method, with the 0 × AA group as the calibration control group (its expression level was set to 1). Independent sample *t*-test was used for comparison between two groups. Different capital letters indicate extremely significant differences compared with the 0 × AA group (*p* < 0.01).

The relative expression levels of *CSN1S1* and *CSN1S2* genes were quantified under the condition of supplementation with the optimal amino acid combination (Figure 5). The results demonstrated that, compared with the 0 × AA group, culture medium supplemented with the optimized ratio of valine, lysine, and threonine significantly enhanced the expression of both *CSN1S1* and *CSN1S2* genes (*p* < 0.01), with *CSN1S1* up-regulated by 2.250-fold and *CSN1S2* by 1.237-fold.

### 3.6 Effect of the optimal ratio of valine, lysine, and threonine supplementation on the expression levels of mTOR signaling pathway-related genes and protein phosphorylation

#### 3.6.1 Effect of the optimal ratio of valine, lysine, and threonine supplementation on the expression levels of Rag small GTPase genes in the upstream region of the mTOR signaling pathway

**Figure 6:**
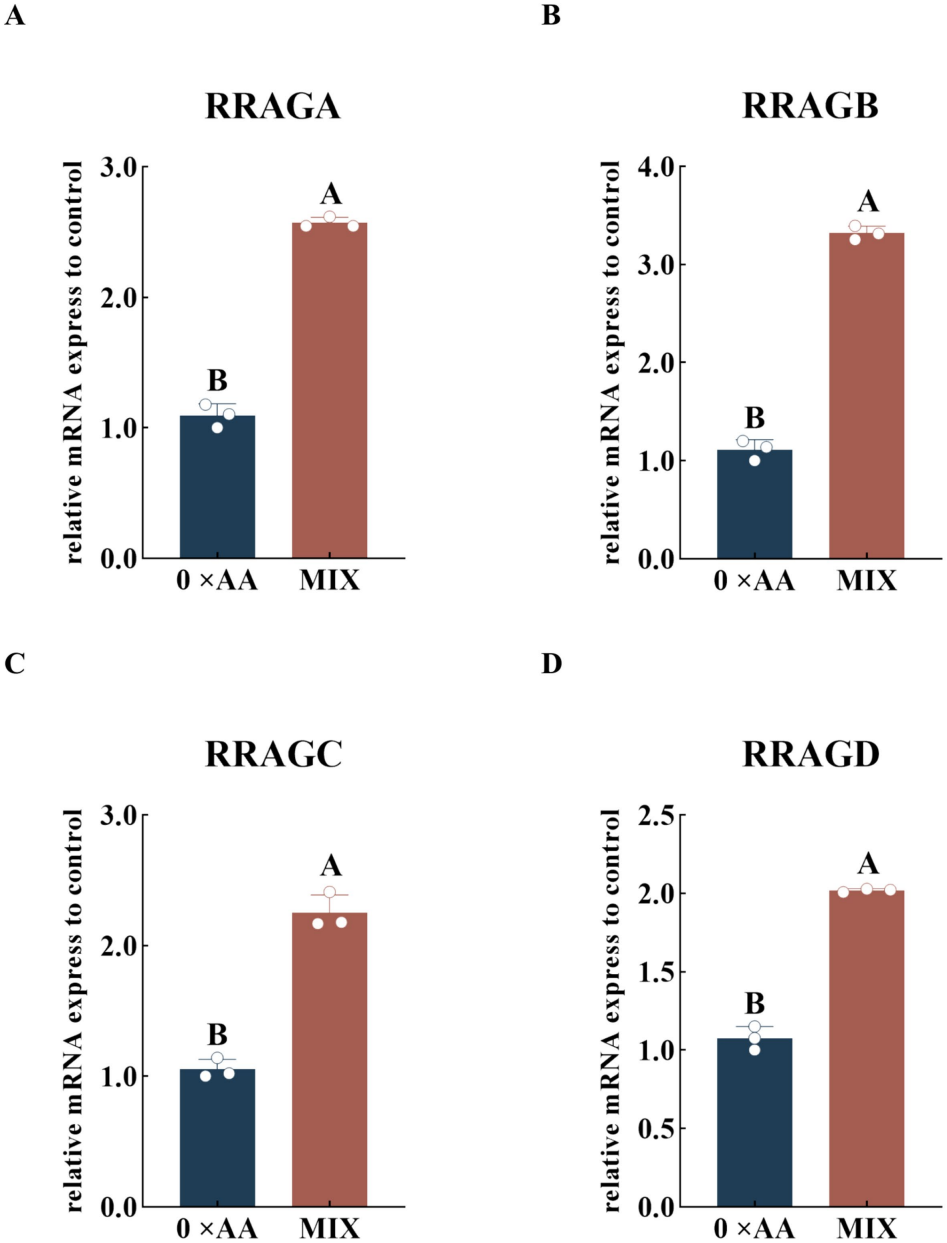
Effect of the optimal ratio of valine, lysine, and threonine supplementation on the relative expression levels of Rag small GTPase-related genes in the upstream region of mTOR. **(A–D)** Respectively represent the relative mRNA expression levels of four protein genes related to the Rag small G proteins upstream of the mTOR signaling pathway (the detection was carried out 12 h after the amino acid supplementation treatment). The error bars represent the SD (*n* = 3). The relative expression levels of genes were calculated using the 2^−ΔΔCt^ method, with the 0 × AA group as the calibration control group (its expression level was set to 1). Independent sample *t*-test was used for comparison between two groups. Different capital letters indicate extremely significant differences compared with the 0 × AA group (*p* < 0.01).

The relative expression levels of upstream Rag small G protein-related genes in the mTOR signaling pathway were quantified under supplementation with the optimal amino acid combination (Figure 6). The results demonstrated that, compared with the 0 × AA group, culture medium supplemented with the optimized ratio of valine, lysine, and threonine significantly enhanced the expression of *RRAGA*, *RRAGB*, *RRAGC*, and *RRAGD* genes (*p* < 0.01). Specifically, *RRAGA* was upregulated 1.351-fold (Figure 6A), *RRAGB* 1.985-fold (Figure 6B), *RRAGC* 1.138-fold (Figure 6C), and *RRAGD* 0.877-fold (Figure 6D).

#### 3.6.2 Effect of the optimal ratio of valine, lysine, and threonine supplementation on the relative expression levels of mTORC1 complex-related genes

**Figure 7:**
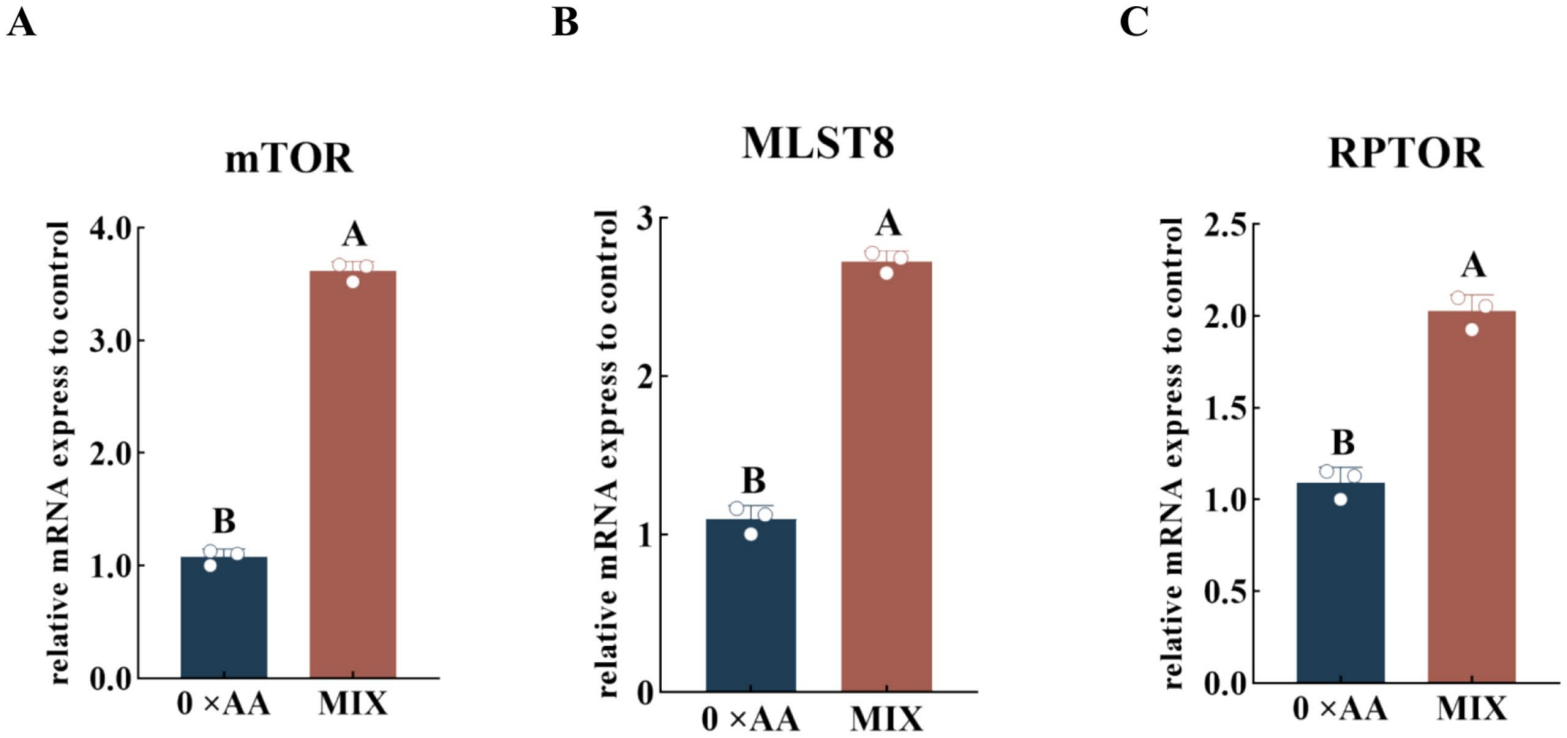
Effects of the optimal molar ratio of valine, lysine, and threonine on the relative mRNA expression levels of genes associated with the mTORC1 complex. **(A–C)** Show the relative mRNA expression levels of the mTORC1 complex-related protein-coding genes *mTOR*, *MLST8*, and *RPTOR*, respectively (the detection was carried out 12 h after the amino acid supplementation treatment). The error bars represent the SD (*n* = 3). The relative expression levels of genes were calculated using the 2^−ΔΔCt^ method, with the 0 × AA group as the calibration control group (its expression level was set to 1). Independent sample *t*-test was used for comparison between two groups. Different capital letters indicate extremely significant differences compared with the 0 × AA group (*p* < 0.01).

The optimal combination of amino acids was supplemented into the MAC-T cell culture medium, and the relative expression levels of mTORC1-related genes upstream in the mTOR signaling pathway were quantitatively assessed (Figure 7). The results demonstrated that, compared with the 0 × AA control, supplementation of the culture medium with the optimal ratio of valine, lysine, and threonine significantly enhanced the relative expression levels of *mTOR*, *MLST8*, and *RPTOR* genes (*p* < 0.01). Specifically, *mTOR* was upregulated by 2.354-fold, *MLST8* by 1.488-fold, and *RPTOR* by 0.852-fold.

#### 3.6.3 Effects of the optimal molar ratio of valine, lysine, and threonine supplementation on the relative mRNA expression levels of downstream target genes in the mTOR signaling pathway

**Figure 8:**
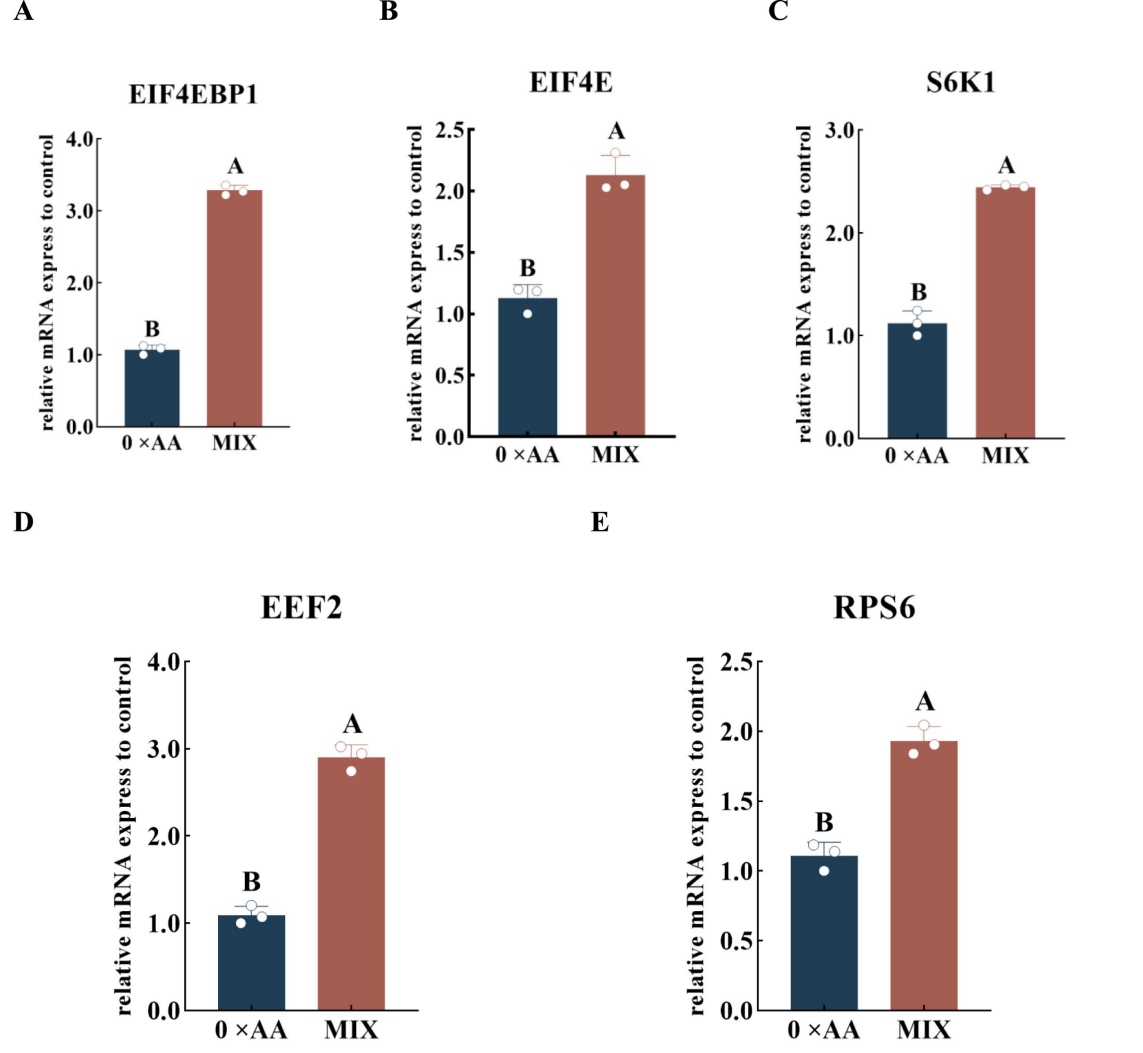
Effects of the optimal molar ratio of valine, lysine, and threonine on the relative mRNA expression levels of downstream genes in the mTOR signaling pathway. **(A–E)** Represent the relative mRNA expression levels of the five key downstream targets of the mTOR pathway: *EIF4EBP1*, *EIF4E*, *S6K1*, *EEF2*, and *RPTOR* (the detection was carried out 12 h after the amino acid supplementation treatment). The error bars represent the SD (*n* = 3). The relative expression levels of genes were calculated using the 2^−ΔΔCt^ method, with the 0 × AA group as the calibration control group (its expression level was set to 1). Independent sample *t*-test was used for comparison between two groups. Different capital letters indicate extremely significant differences compared with the 0 × AA group (*p* < 0.01).

The optimal amino acid combination was supplemented into the MAC-T cell culture medium, and the relative expression levels of downstream genes in the mTOR signaling pathway were quantitatively assessed (Figure 8). The results showed that compared with 0 × AA, the optimal ratio amino acid mixture medium with valine, lysine and threonine addition significantly increased the relative expression levels of *EIF4EBP1*, *EIF4E*, *S6K1*, *EEF2* and *RPS6* genes (*p* < 0.01). Among them, *EIF4EBP1* was up-regulated by 2.064 times, *EIF4E* by 0.888 times, *S6K1* by 1.180 times, *EEF2* by 1.660 times, and *RPS6* by 0.741 times.

#### 3.6.4 Effects of the optimal molar ratio of valine, lysine, and threonine supplementation on the phosphorylation levels of downstream target proteins in the mTOR signaling pathway

**Figure 9:**
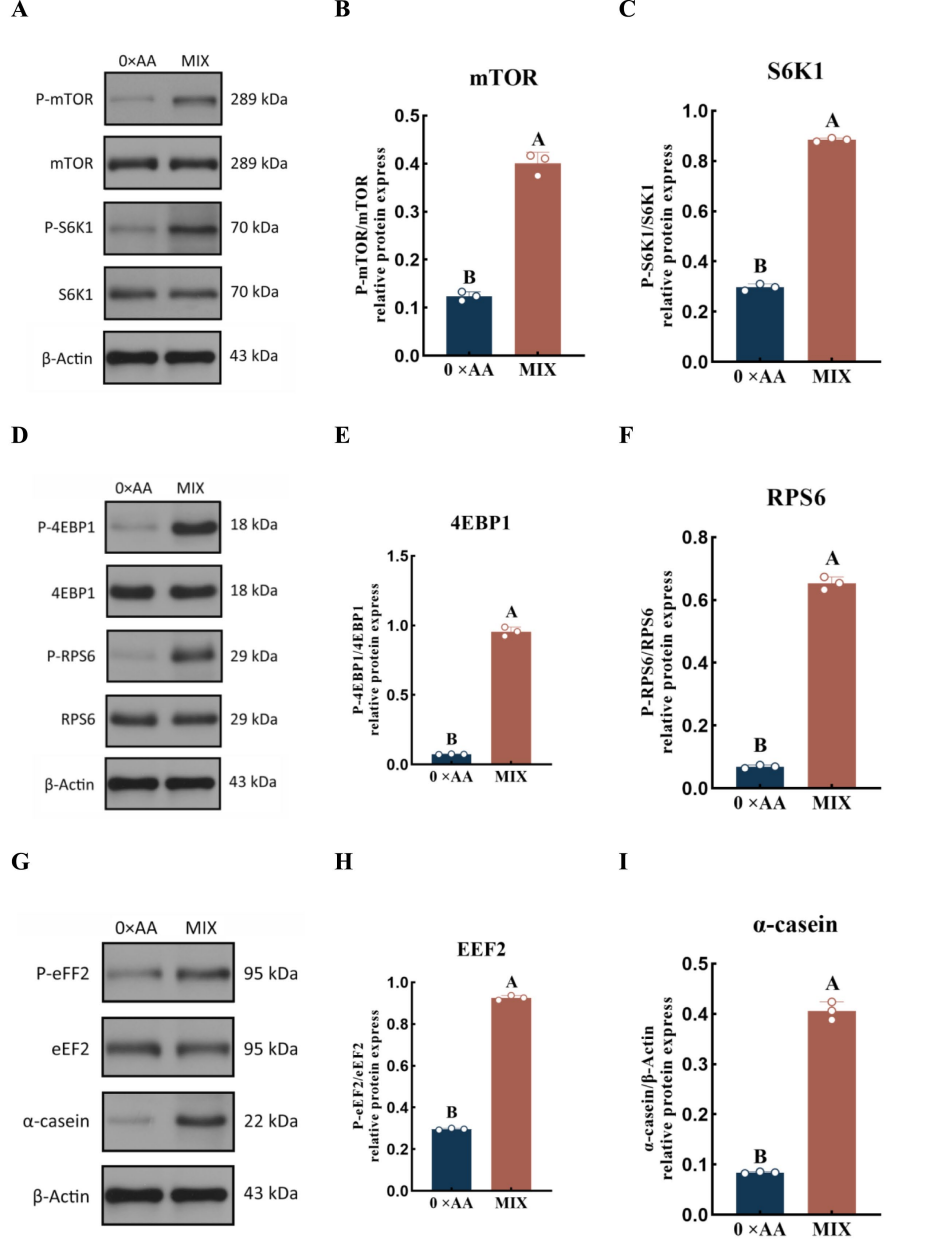
Effects of the optimal molar ratio of valine, lysine, and threonine on the phosphorylation levels of downstream proteins in the mTOR signaling pathway. **(A)** The protein phosphorylation levels of phosphorylated mTOR and S6K1 were detected by western blotting. **(B)** The phosphorylation level of mTOR protein (P-mTOR/mTOR) was quantified and calculated using β-actin and mTOR as internal references. **(C)** The phosphorylation level of S6K1 protein (P-S6K1/S6K1) was quantified and calculated using β-actin and S6K1 as internal references. **(D)** The protein phosphorylation levels of phosphorylated 4EBP1 and RPS6 were detected by Western blotting. **(E)** The phosphorylation level of 4EBP1 protein (P-4EBP1/4EBP1) was quantified and calculated using β-actin and 4EBP1 as internal references. **(F)** The phosphorylation level of RPS6 protein (P-RPS6/RPS6) was quantified and calculated using β-actin and RPS6 as internal references. **(G)** The protein phosphorylation level of phosphorylated EEF2 and the amount of α-casein were detected by Western blotting. **(H)** The phosphorylation level of EEF2 protein (P-EEF2/EEF2) was quantified and calculated using β-actin and EEF2 as internal references. **(I)** The amount of α-casein was detected by estern blotting (he detection was carried out 12 h after the amino acid supplementation treatment). The error bars represent the SD (*n* = 3). Independent sample *t*-test was used for comparison between two groups. Different capital letters indicate extremely significant differences compared with the 0 × AA group (*p* < 0.01).

The above results indicate that supplementation with the optimal combination of amino acids in the MAC-T cell culture medium enhances the expression of genes associated with the mTOR signaling pathway. To further validate activation of this pathway and its role in α-casein synthesis, we assessed the phosphorylation levels of key mTOR pathway proteins and α-casein abundance in both the optimal combination amino acid group and the 0 × AA control group (Figure 9). The results showed that, compared with the 0 × AA control, supplementation with the optimal ratio of valine, lysine, and threonine in the amino acid mixture significantly increased the phosphorylation levels of mTOR, S6K1, 4EBP1, RPS6, and eEF2 proteins, as well as the synthesis of α-casein (*p* < 0.01). Specifically, phosphorylation of mTOR was upregulated by 7.330-fold, S6K1 by 1.977-fold, 4EBP1 by 11.197-fold, RPS6 by 8.487-fold, and eEF2 by 2.123-fold, while α-casein synthesis increased by 3.831-fold.

### 3.7 Inhibition of the mTOR signaling pathway to validate its involvement in mediating α-casein synthesis in MAC-T cells induced by the optimal combination of valine, lysine, and threonine

#### 3.7.1 Inhibition of the mTOR signaling pathway was employed to validate its functional involvement in up-regulating the expression of the α-casein encoding gene induced by the optimal combination of valine, lysine, and threonine

**Figure 10:**
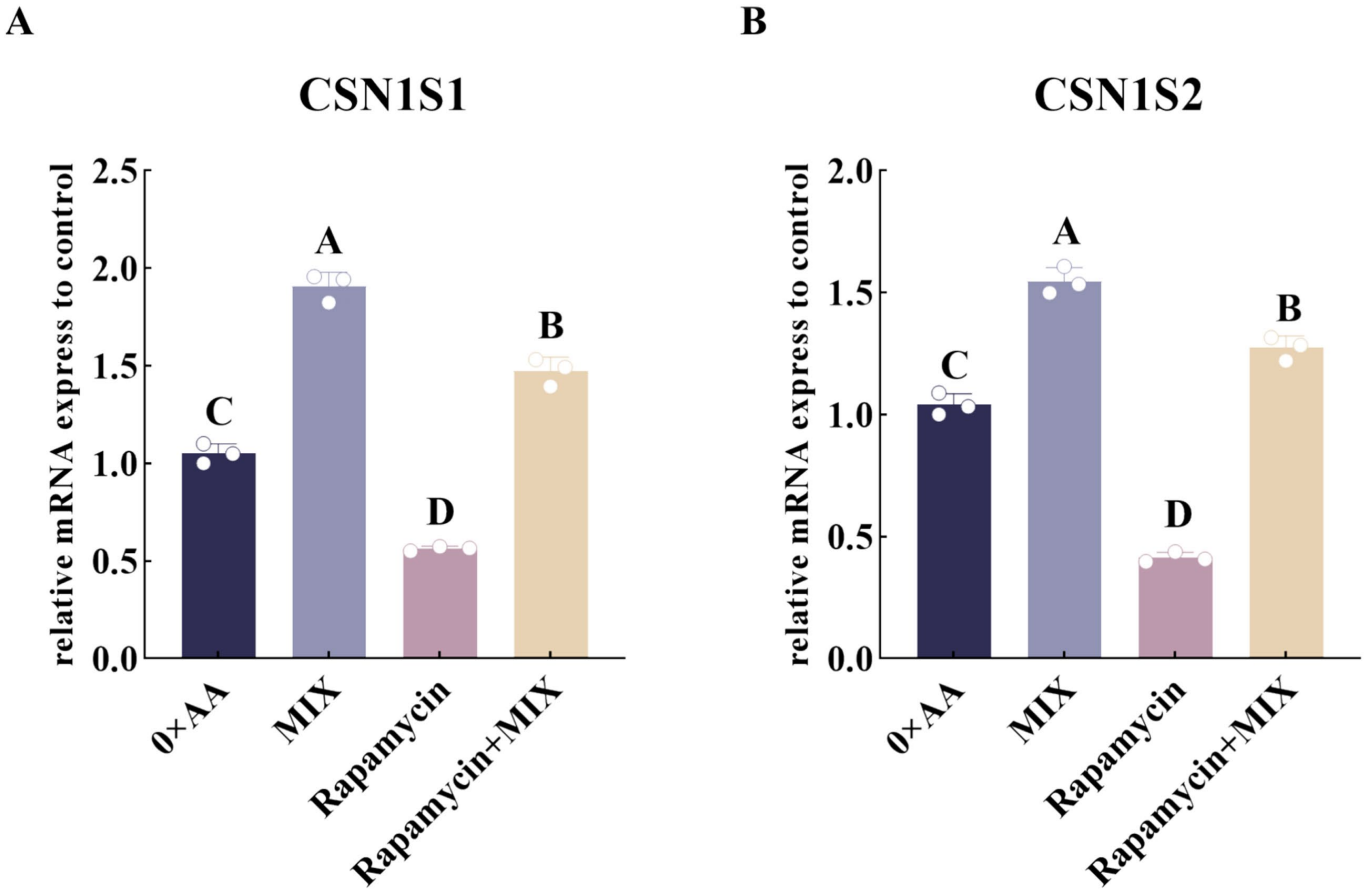
Inhibition of the mTOR signaling pathway to validate its involvement in up-regulating the expression of the α-casein encoding gene induced by the optimal combination of valine, lysine, and threonine. **(A,B)** Respectively represent the relative expression levels of the *CSN1S1* and *CSN1S2* genes in different groups (the detection was carried out 12 h after the amino acid supplementation treatment). The error bars represent the SD (*n* = 3). Data were analyzed by one-way ANOVA, and the *post hoc* multiple comparisons were conducted using Duncan’s new multiple range test. Different lowercase letters in the figures indicate significant differences (*p* < 0.05), and different uppercase letters indicate extremely significant differences (*p* < 0.01).

The above results suggest that the optimal combination of amino acids promotes α-casein synthesis through activation of the mTOR signaling pathway. To test this hypothesis, rapamycin was applied to selected experimental groups, and the relative mRNA expression levels of *CSN1S1* and *CSN1S2*—genes directly involved in α-casein synthesis—were quantified (Figure 10). The results showed that, compared with the 0 × AA group, supplementation of rapamycin into the MAC-T culture medium significantly downregulated the relative mRNA expression levels of *CSN1S1* and *CSN1S2* by 0.705-fold and 0.732-fold, respectively (*p* < 0.01). However, co-treatment with the optimal mixture of valine, lysine, and threonine (8:2:1) partially reversed this suppression. Specifically, compared to the rapamycin-only group, the addition of the three amino acids at the optimal ratio (8:2:1) significantly restored the expression levels of *CSN1S1* and *CSN1S2* by 1.615-fold and 2.074-fold, respectively (*p* < 0.01), indicating a rescue effect on α-casein gene expression.

#### 3.7.2 Inhibition of the mTOR signaling pathway was employed to validate its involvement in up-regulating the expression of Rag small GTPase genes located upstream of the mTOR signaling cascade under the optimal combination of valine, lysine, and threonine

**Figure 11:**
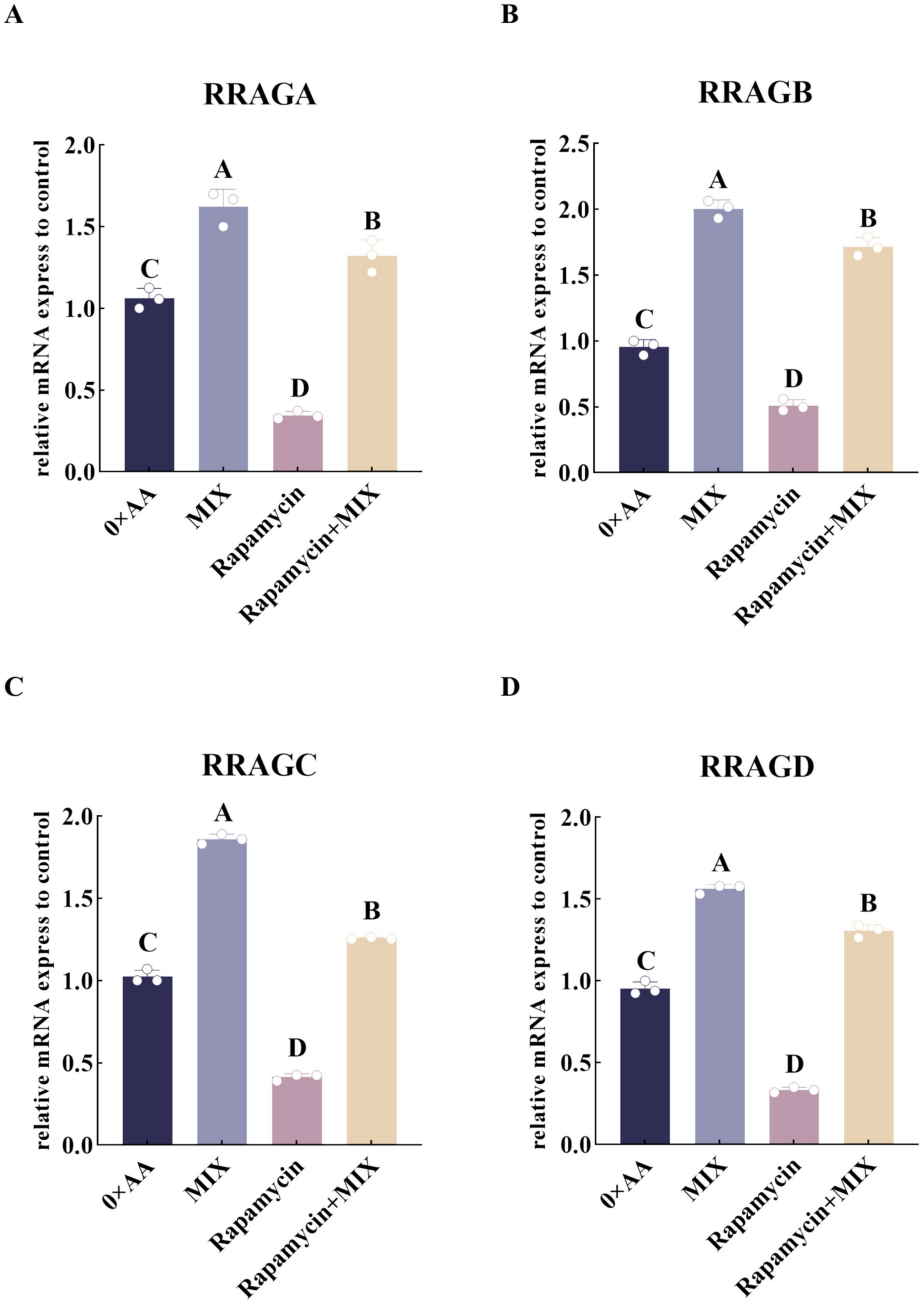
Inhibition of the mTOR signaling pathway to validate its involvement in up-regulating the expression of Rag small GTPase genes located upstream of the mTOR cascade under the optimal combination of valine, lysine, and threonine. **(A–D)** Respectively represent the relative expression levels of Rag small G protein-related genes in different groups (the detection was carried out 12 h after the amino acid supplementation treatment). The error bars represent the SD (*n* = 3). Data were analyzed by one-way ANOVA, and the post hoc multiple comparisons were conducted using Duncan’s new multiple range test. Different lowercase letters in the figures indicate significant differences (*p* < 0.05), and different uppercase letters indicate extremely significant differences (*p* < 0.01).

To verify that the optimal combination of amino acids can promote the synthesis of α-casein by activating the mTOR signaling pathway, rapamycin was added to the selected experimental groups, and the relative expression levels of upstream Rag small G protein-related genes in the mTOR signaling pathway were quantitatively analyzed. The results showed that, compared with the 0 × AA group, supplementation of rapamycin into the MAC-T culture medium significantly downregulated the relative mRNA expression of *RRAGA*, *RRAGB*, *RRAGC*, and *RRAGD* (*p* < 0.01). However, co-treatment with the optimal mixture of valine, lysine, and threonine (8:2:1) partially reversed this suppression. Specifically, compared to the rapamycin-only group, the addition of the three amino acids at the optimal ratio (8:2:1) significantly restored the mRNA expression levels of *RRAGA* (2.819-fold), *RRAGB* (2.364-fold), *RRAGC* (2.036-fold), and *RRAGD* (2.926-fold) (*p* < 0.01), indicating a rescue effect on the upstream regulators of the mTOR signaling pathway (Figure 11).

#### 3.7.3 Inhibition of the mTOR signaling pathway was employed to validate its role in up-regulating the expression of mTORC1 complex-associated genes under the optimal combination of valine, lysine, and threonine

**Figure 12:**
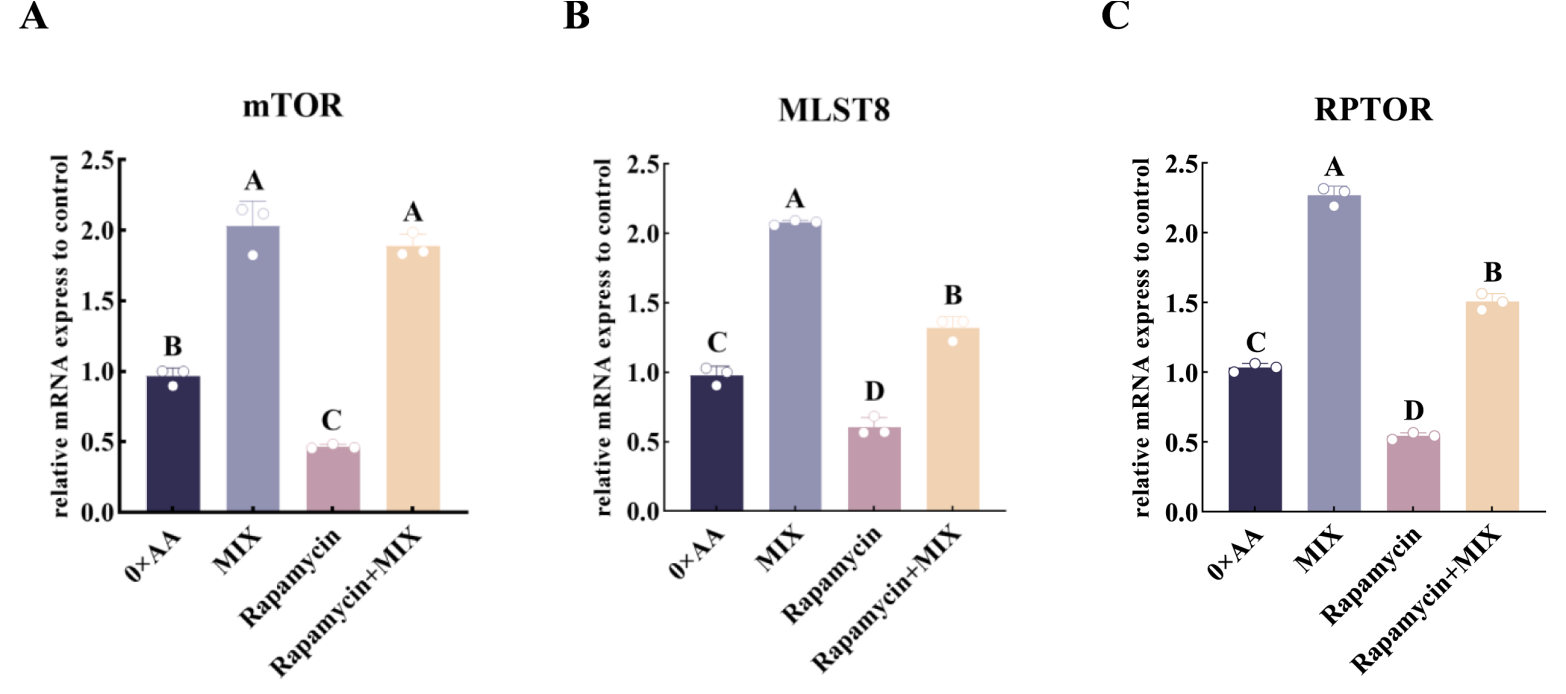
Inhibition of the mTOR signaling pathway to validate its role in up-regulating the expression of mTORC1 complex-associated genes under the optimal combination of valine, lysine, and threonine. **(A–C)** Respectively represent the relative expression levels of mTORC1-related protein genes (*mTOR*, *MLST8, RPTOR*) in different groups (the detection was carried out 12 h after the amino acid supplementation treatment). 0 × AA group: serum-free DMEM basal medium. MIX group: the optimal combination of amino acids (valine 36.114 mmol/L + lysine 9.027 mmol/L + threonine 4.602 mmol/L) was added to the basic medium. Rapamycin group: add 100 nmol/L rapamycin to the DMEM basal medium. Rapamycin+MIX group: the basic culture medium was supplemented with 100 nmol/L rapamycin and the optimal combination of amino acids simultaneously. Data were analyzed by one-way ANOVA, and the post hoc multiple comparisons were conducted using Duncan’s new multiple range test. Different lowercase letters in the figures indicate significant differences (*p* < 0.05), and different uppercase letters indicate extremely significant differences (*p* < 0.01).

To verify whether the optimal combination of amino acids could promote the synthesis of α-casein by activating the mTOR signaling pathway, rapamycin was added to the selected experimental groups, and the relative expression levels of mTORC1-related protein genes were quantitatively analyzed. The results showed that, compared with the 0 × AA group, supplementation of rapamycin into the MAC-T cell culture medium significantly suppressed the relative mRNA expression of *mTOR*, *MLST8*, and *RPTOR* (*p* < 0.01). However, co-treatment with the optimal ratio mixture of valine, lysine, and threonine partially reversed this suppression. Specifically, compared to the rapamycin-only group, the addition of the three amino acids in their optimal combination led to a significant upregulation of *mTOR* (3.052-fold), *MLST8* (1.171-fold), and *RPTOR* (1.777-fold) mRNA expression levels (*p* < 0.01) (Figure 12).

#### 3.7.4 Inhibition of the mTOR signaling pathway to validate its role in up-regulating the expression of downstream target genes under the optimal combination of valine, lysine, and threonine

**Figure 13:**
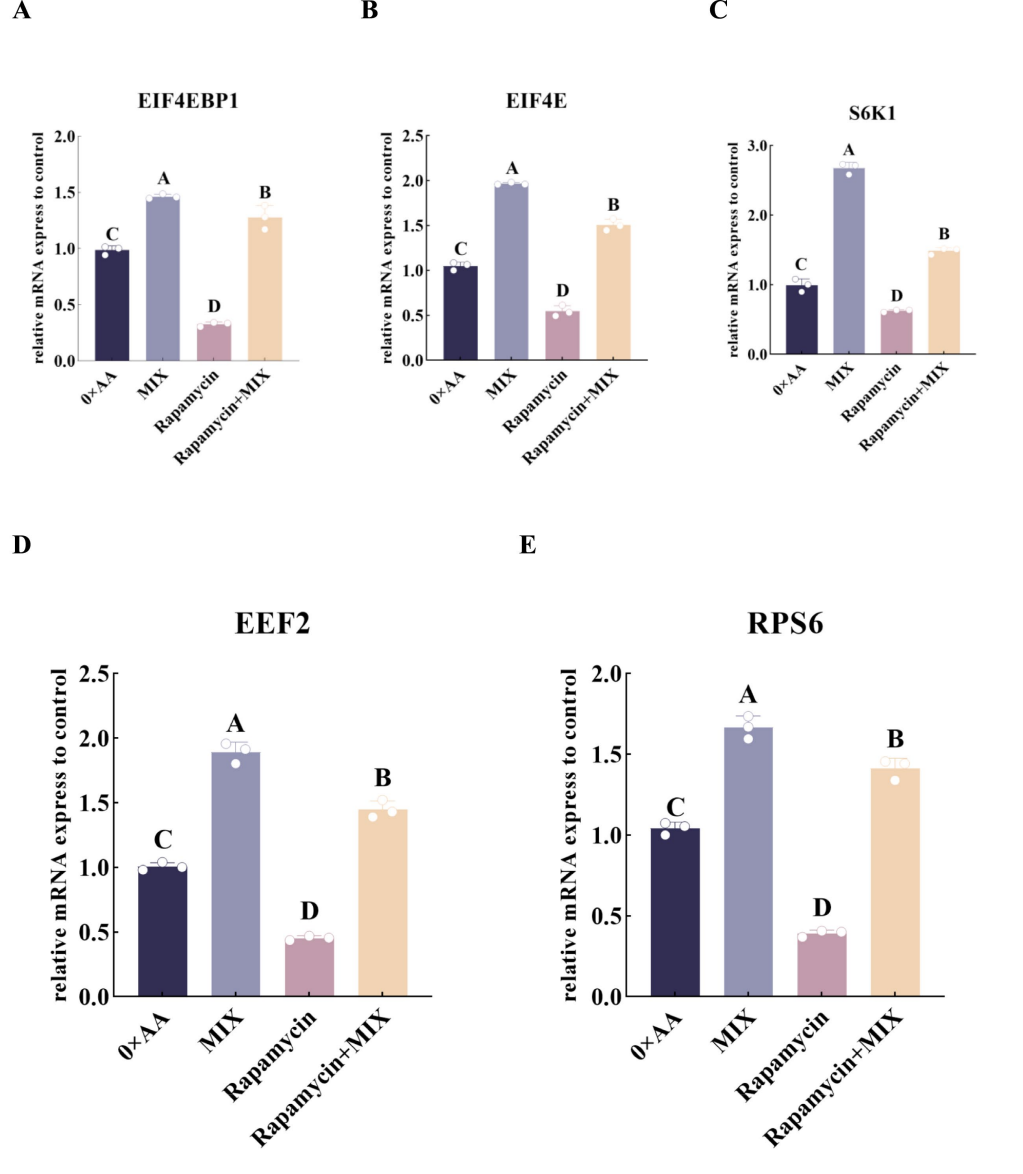
Inhibition of the mTOR signaling pathway to validate its role in up-regulating the expression of downstream target genes under the optimal combination of valine, lysine, and threonine. **(A–E)** Respectively represent the relative expression levels of key downstream protein genes of the mTOR signaling pathway (EIF4EBP1, EIF4E, S6K1, EEF2, and RPS6) in different groups (the detection was conducted 12 h after amino acid supplementation treatment). 0 × AA group: serum-free DMEM basal medium. MIX group: the optimal combination of amino acids (valine 36.114 mmol/L + lysine 9.027 mmol/L + threonine 4.602 mmol/L) was added to the basic medium. Rapamycin group: add 100 nmol/L rapamycin to the DMEM basal medium. Rapamycin+MIX group: the basic culture medium was supplemented with 100 nmol/L rapamycin and the optimal combination of amino acids simultaneously. Data were analyzed by one-way ANOVA, and the post hoc multiple comparisons were conducted using Duncan’s new multiple range test. Different lowercase letters in the figures indicate significant differences (*p* < 0.05), and different uppercase letters indicate extremely significant differences (*p* < 0.01).

To verify whether the optimal combination of amino acids can promote the synthesis of α-casein by activating the mTOR signaling pathway, rapamycin was added to the selected experimental groups, and the relative expression levels of key protein genes downstream of the mTOR signaling pathway were quantitatively analyzed. The results demonstrated that, compared with the 0 × AA group, supplementation of rapamycin into the MAC-T culture medium significantly suppressed the relative mRNA expression of key translational regulatory genes, including *EIF4EBP1*, *EIF4E*, *S6K1*, *EEF2*, and *RPS6* (*p* < 0.01). However, co-treatment with the optimal combination of valine, lysine, and threonine in the rapamycin-containing medium partially reversed this inhibitory effect. Specifically, compared to the rapamycin-only group, the addition of the three amino acids at their optimal ratio led to a significant up-regulation of *EIF4EBP1* (2.914-fold increase), *EIF4E* (1.753-fold increase), *S6K1* (1.373-fold increase), *EEF2* (2.191-fold increase), and *RPS6* (2.603-fold increase) (*p* < 0.01), indicating an attenuation of rapamycin-induced suppression (Figure 13).

#### 3.7.5 Inhibition of the mTOR signaling pathway to validate its role in promoting phosphorylation of downstream target proteins under the optimal combination of valine, lysine, and threonine

**Figure 14:**
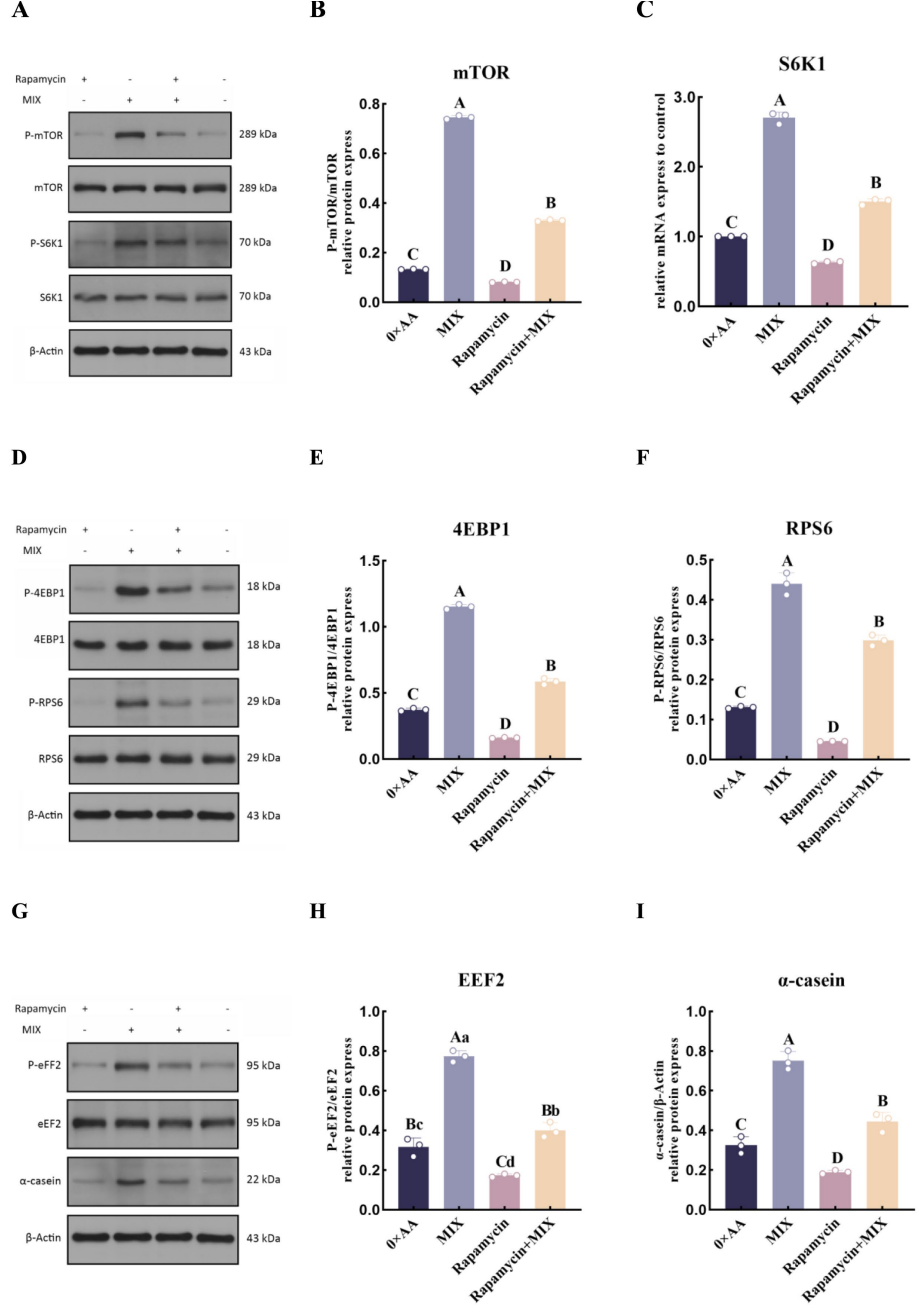
Inhibition of the mTOR signaling pathway to validate its role in promoting phosphorylation of downstream target proteins under the optimal combination of valine, lysine, and threonine. **(A)** The protein phosphorylation levels of phosphorylated mTOR and S6K1 were detected by western blotting. **(B)** The phosphorylation level of mTOR protein (P-mTOR/mTOR) was quantified and calculated using β-actin and mTOR as internal references. **(C)** The phosphorylation level of S6K1 protein (P-S6K1/S6K1) was quantified and calculated using β-actin and S6K1 as internal references. **(D)** The protein phosphorylation levels of phosphorylated 4EBP1 and RPS6 were detected by western blotting. **(E)** The phosphorylation level of 4EBP1 protein (P-4EBP1/4EBP1) was quantified and calculated using β-actin and 4EBP1 as internal references. **(F)** The phosphorylation level of RPS6 protein (P-RPS6/RPS6) was quantified and calculated using β-actin and RPS6 as internal references. **(G)** The protein phosphorylation level of phosphorylated EEF2 and the amount of α-casein were detected by western blotting. **(H)** The phosphorylation level of EEF2 protein (P-EEF2/EEF2) was quantified and calculated using β-actin and EEF2 as internal references. **(I)** The amount of α-casein was detected by western blotting (the detection was carried out 12 h after the amino acid supplementation treatment). The error bars represent the SD (*n* = 3). Data were analyzed by one-way ANOVA, and the post hoc multiple comparisons were conducted using Duncan’s new multiple range test. Different lowercase letters in the figures indicate significant differences (*p* < 0.05), and different uppercase letters indicate extremely significant differences (*p* < 0.01).

To verify whether the optimal combination of amino acids could promote the synthesis of α-casein by activating the mTOR signaling pathway, rapamycin was added to the selected experimental groups, and the phosphorylation levels of key proteins in the mTOR signaling pathway and the synthesis of α-casein were quantitatively analyzed. The results demonstrated that, compared with the 0 × AA group, supplementation of rapamycin into the MAC-T culture medium significantly suppressed the phosphorylation levels of mTOR (Ser2448), S6K1 (Thr389/Thr412), 4EBP1 (Thr45), RPS6 (Ser235), and eEF2 (Tyr443) proteins (*p* < 0.01). However, co-treatment with the optimal mixture of valine, lysine, and threonine partially reversed this suppression. Specifically, relative to the rapamycin-only group, the addition of the three amino acids at their optimal ratio led to a significant up-regulation in the phosphorylation of mTOR (3.073-fold), S6K1 (3.007-fold), 4EBP1 (2.652-fold), RPS6 (5.603-fold), and eEF2 (1.319-fold), as well as an increase in α-casein synthesis (1.558-fold) (*p* < 0.01) (Figure 14).

## 4 Discussion

### 4.1 Effects of individual supplementation with valine, lysine, or threonine and the optimal amino acid combination on α-casein synthesis in MAC-T cells

Valine, lysine, and threonine—key essential amino acids for casein synthesis—each exert a significant regulatory effect on α-casein synthesis in MAC-T cells when supplemented individually (6, 10, 11). However, increasing amino acid concentrations in the MAC-T cell culture medium does not lead to unlimited enhancement of α-casein production. Excessive supplementation fails to further promote α-casein synthesis and may even suppress it (5). In this study, individual addition of valine, lysine, or threonine resulted in a biphasic response: intracellular α-casein synthesis first increased and then decreased with escalating amino acid levels. Peak synthesis was observed at 4 × Val, 1 × Lys, and 0.5 × Thr, respectively. Notably, when lysine and threonine levels exceeded 4 × Lys and 8 × Thr, α-casein synthesis was significantly inhibited, demonstrating a clear dose-dependent effect of each amino acid on protein synthesis. At low amino acid concentrations, MAC-T cells efficiently utilize available substrates for α-casein production, with maximal stimulation achieved at the aforementioned optimal levels. However, as amino acid levels rise to excess, intracellular pools become saturated and amino acid transporters become overloaded, leading to reduced utilization efficiency (12). To overcome this limitation and maximize α-casein synthesis, we investigated the optimal combination of the three amino acids. The MIX group, formulated using response surface methodology, exhibited significantly higher α-casein synthesis than both the individually optimized groups and the 0 × AA control, highlighting the irreplaceable synergistic effect among the three amino acids. Previous studies have shown that excessive levels of valine, lysine, or threonine can disrupt amino acid homeostasis, impair the transport of other amino acids, and consequently inhibit α-casein synthesis (13–15). Importantly, the MIX formulation—through precise stoichiometric balancing (Val: Lys: Thr = 8:2:1)—effectively circumvented these inhibitory effects, thereby maximizing α-casein output. These findings demonstrate that the synergistic interaction of combined amino acids enhances amino acid uptake efficiency and overcomes the dosage constraints associated with individual supplementation, ultimately improving α-casein synthesis efficiency.

### 4.2 Effects of supplementing the optimal combination of valine, lysine, and threonine on the relative mRNA expression levels of the α-casein-encoding gene in MAC-T cells

Amino acids serve not only as substrates for α-casein synthesis but also as critical signaling and regulatory molecules (16). Wang (17) reported that lysine and threonine are key limiting amino acids for milk protein synthesis, and their deficiency significantly reduces casein gene expression in mammary epithelial cells. In this study, the addition of valine further optimizes amino acid balance, thereby enhancing gene expression efficiency. Zhang et al. (18) demonstrated that valine, as a branched-chain amino acid (BCAA), promotes protein translation through activation of the mTOR signaling pathway. Lysine and threonine not only activate the mTOR pathway to stimulate translation but also modulate cellular metabolic status, providing sufficient substrates and energy to support the expression of casein-related genes (19, 20). Our results show that supplementing the culture medium with the optimal combination of valine, lysine, and threonine significantly increases the relative mRNA expression levels of the α-casein encoding genes *CSN1S1* and *CSN1S2*. Compared with the serum-free control medium (0 × AA), the expression levels of *CSN1S1* and *CSN1S2* were up-regulated by 2.250-fold and 1.237-fold, respectively, with the increase in *CSN1S1* expression being significantly greater than that of *CSN1S2*. This difference may result from the synergistic effects of the three essential amino acids, which regulate α-casein synthesis through modulation of transcriptional activity and signaling pathways. Additionally, the promoter regions of *CSN1S1* and *CSN1S2* may differ in their responsiveness to amino acid signaling (21), potentially reflecting the dominant role of αs1-casein in milk protein composition. These findings indicate that the optimal combination of valine, lysine, and threonine enhances α-casein synthesis by significantly up-regulating the expression of *CSN1S1* and *CSN1S2*, highlighting the importance of coordinated amino acid supplementation in regulating milk protein gene expression.

### 4.3 Effects of supplementing the optimal combination of valine, lysine, and threonine on the expression and phosphorylation status of mTOR pathway-associated genes involved in α-casein synthesis

Amino acids function not only as building blocks but also as signaling molecules that regulate milk protein translation initiation and ribosome biogenesis through the mammalian target of rapamycin complex 1 (mTORC1) signaling pathway, positioning them as central regulators of milk protein synthesis (22). Kim (23) demonstrated that supplementing a single amino acid can elevate the relative mRNA expression and protein phosphorylation levels of genes associated with the mTOR signaling pathway. However, due to competition among amino acids for shared transport systems, the stimulatory effects on gene expression and phosphorylation are often limited. Tan and Miyamoto (24) reported that Rag small GTPases serve as critical mediators for recruiting mTORC1 complexes to the lysosomal membrane; increased expression of Rag-related genes promotes mTORC1 localization and activation, thereby initiating downstream signaling cascades essential for α-casein synthesis. The mTORC1 complex is primarily composed of three core components: mTOR, GβL (also known as mLST8), and Raptor. As the catalytic kinase subunit, upregulation of mTOR expression expands the pool of active kinases. GβL stabilizes the structural integrity of mTOR, while Raptor facilitates substrate recruitment by recognizing the TOS motif in downstream effectors such as 4EBP1 and S6K1 (25, 26). This coordinated upregulation enhances both the assembly efficiency and functional abundance of the mTORC1 complex, potentially amplifying its capacity to transmit signals to downstream targets (27). In this study, supplementation with the optimal combination of valine, lysine, and threonine significantly upregulated the expression of upstream Rag GTPase-related genes (*RRAGA*, *RRAGB*, *RRAGC*, and *RRAGD*) as well as mTORC1 core component genes (*mTOR*, *MLST8*, and *RPTOR*). Notably, the fold-increase in mTOR protein phosphorylation was substantially greater than that of *mTOR* gene expression (7.330-fold vs. 2.354-fold). This discrepancy suggests that the synergistic action of Val, Lys, and Thr not only enhances transcriptional activity of key regulatory genes—including Rag GTPases—but also potentiates post-translational activation of mTOR. By optimizing intracellular signal transduction, this amino acid combination may facilitate more efficient phosphorylation of mTOR during post-translational modification, resulting in a disproportionately higher increase in phosphorylated mTOR levels compared to its mRNA expression.

Quan et al. (28) demonstrated that supplementation with a single amino acid can modulate the phosphorylation of downstream mTORC1 targets, including 4EBP1 and S6K1, thereby regulating milk protein synthesis. In this study, the optimal combination of valine, lysine, and threonine in the mixed culture medium significantly upregulated the relative mRNA expression levels of key downstream genes in the mTOR signaling pathway (*mTOR*, *EIF4EBP1*, *EIF4E*, *S6K1*, *EEF2*, and *RPS6*), enhanced the phosphorylation levels of corresponding proteins (mTOR, 4EBP1, S6K1, eEF2, and RPS6), and increased α-casein synthesis. Notably, the relative expression of *EIF4EBP1* was elevated by 2.064-fold, while its protein phosphorylation level increased dramatically by 11.197-fold, and *EIF4E* gene expression rose by 0.888-fold. This may be attributed to MAC-T cells, upon exposure to the optimal amino acid combination, undergoing extensive phosphorylation of 4EBP1, leading to its dissociation from eIF4E. The released eIF4E subsequently associates with other translation initiation factors to form the eIF4F complex, thereby facilitating translation initiation and promoting α-casein synthesis (29). Furthermore, the combination treatment significantly enhanced both the expression and phosphorylation of S6K1, RPS6, and EEF2, coinciding with a marked increase in α-casein production. These findings suggest that the optimal valine, lysine, and threonine formulation activates S6K1 through phosphorylation, which in turn stimulates downstream effectors such as RPS6 and eEF2, thus promoting the initiation of α-casein mRNA translation (30). Chen (31) reported that phosphorylation of eEF2 typically suppresses α-casein synthesis by inhibiting translational elongation. However, in this study, despite significant upregulation of both *EEF2* gene expression and protein phosphorylation, α-casein synthesis was still markedly enhanced. This apparent contradiction may be explained by the robust activation of the mTOR signaling pathway: the phosphorylation level of mTOR protein increased by 7.330-fold, strongly driving the phosphorylation of S6K1 and RPS6, enhancing ribosomal biogenesis and translation initiation efficiency, and creating a highly favorable intracellular environment for protein synthesis. Although elevated eEF2 phosphorylation may transiently slow translation elongation, the dominant anabolic drive mediated by mTOR overrides this inhibitory signal, resulting in a net increase in global protein synthesis capacity. Collectively, these results indicate that supplementing MAC-T cell culture medium with the optimal combination of valine, lysine, and threonine potently enhances the expression of upstream Rag GTPase-related genes, promotes mTOR phosphorylation, and subsequently activates downstream signaling cascades—ultimately leading to significantly increased α-casein synthesis.

### 4.4 Role of mTOR signaling pathway inhibition in mediating α-casein synthesis by MAC-T cells under the optimal combination of valine, lysine, and threonine

The mTOR signaling pathway serves as a central regulator of casein synthesis, and valine, lysine, and threonine have all been shown to modulate protein synthesis through this pathway (32, 33). In recent years, the impact of amino acid ratios on mTOR signaling and downstream protein synthesis has emerged as a key research focus (34). Previous findings indicate that supplementing culture medium with the optimal combination of valine, lysine, and threonine (8:2:1) enhances α-casein synthesis in MAC-T cells by activating the mTOR signaling cascade. To further investigate the functional role of the mTOR pathway in mediating α-casein production under this optimal amino acid formulation, rapamycin was applied to specifically inhibit mTOR phosphorylation. Subsequently, changes in the expression of α-casein-encoding genes (*CSN1S1* and *CSN1S2*), mTOR pathway-related genes, downstream effector proteins and their phosphorylation status, as well as α-casein synthesis levels, were systematically evaluated. Results showed that treatment with rapamycin alone significantly suppressed the expression of both *CSN1S1* and *CSN1S2* genes. However, co-supplementation with the optimal amino acid combination partially reversed this suppression, restoring gene expression by 1.615-fold and 2.074-fold, respectively—indicating that the tri-amino acid formulation promotes transcriptional activity via an mTOR-dependent mechanism (4). Furthermore, compared to the rapamycin-treated group, the addition of the optimal amino acid mixture markedly upregulated the relative mRNA expression of Rag GTPase-related genes (*RRAGA*, *RRAGB*, *RRAGC*, *RRAGD*) and mTORC1 core components (*mTOR*, *MLST8*, *RPTOR*), along with enhancing the phosphorylation levels of downstream signaling molecules. Notably, *mTOR* gene expression was significantly increased and nearly restored to the level observed in the MIX (positive control) group. This might be because amino acids can regulate protein synthesis through mTORC1-independent pathways, including the GCN2/eIF2α pathway, FGF21 signaling, and direct regulation by intracellular amino acid-sensing proteins. The partial recovery effect observed in this study may involve compensatory activation of these pathways (35). Additionally, eEF2 phosphorylation was elevated in cells treated with the amino acid mixture compared to the 0 × AA (amino acid-deficient) group. Since phosphorylated eEF2 inhibits peptide chain elongation and typically suppresses translation, this finding appears counterintuitive. Nevertheless, the magnitude of eEF2 phosphorylation increase was substantially lower than the enhancements observed in mTOR, S6K1, 4EBP1, and RPS6 phosphorylation, as well as in overall α-casein synthesis. Thus, despite partial inhibition of elongation, the net effect was a significant increase in α-casein production, driven primarily by robust activation of mTOR-mediated translation initiation and ribosomal biogenesis. All the above results indicate that the addition of Rapamycin can inhibit the mTOR signaling pathway and the synthesis of α-casein, but this inhibitory effect has limitations (36). This may be because Rapamycin mainly inhibits the kinase activity of mTORC1 by binding to FKBP12 to form a complex, but the inhibition of the mTORC1 complex and the phosphorylation of certain substrates is limited (37), and the super-physiological level of amino acid concentration can partially counteract the inhibitory effect of Rapamycin by enhancing amino acid signal input. The optimal combination concentration used in this study is much higher than the physiological level of bovine plasma. This high concentration of amino acids may maintain a certain signal output under partial inhibition by enhancing the activity of Rag GTPase and promoting the recruitment of mTORC1 to the lysosomal membrane (16). The addition of the optimal combination of valine, lysine, and threonine helps mediate the synthesis of α-casein in MAC-T cells through the activation of the mTOR signaling pathway.

The optimal amino acid combination levels identified in this study (valine: 36.114 mmol/L, lysine: 9.027 mmol/L, and threonine: 4.602 mmol/L) are far higher than the physiological levels in bovine plasma. This difference objectively reflects the essential distinction between *in vitro* culture systems and *in vivo* physiological environments: *in vitro* cultured cells are directly exposed to the culture medium, lacking the continuous supply of blood circulation, the buffering effect of plasma proteins, and the metabolic regulation of organs such as the liver and small intestine (5). Therefore, higher extracellular nutrient concentrations are often required to achieve comparable metabolic responses to those *in vivo*. Against this backdrop, the core value of the optimal combination (8:2:1) determined in this study lies in providing the ratio among the three amino acids rather than their absolute concentrations. This ratio reflects the synergistic demand of α-casein synthesis for the three amino acids and can provide a theoretical basis for optimizing the amino acid balance in the diet. Additionally, the study found that valine, lysine, and threonine all have a dose-dependent effect on α-casein synthesis in MAC-T cells, providing a direction for subsequent *in vivo* dose optimization rather than directly applying the concentrations. Further *in vivo* dose titration experiments are needed to explore the appropriate addition levels within the physiological range of dairy cows while maintaining this ratio, and to verify this through plasma amino acid profile analysis, providing a scientific basis for the formulation of precise nutrition strategies.

## 5 Conclusion

Valine, lysine, and threonine each effectively enhance α-casein synthesis in MAC-T cells. The optimal effect—maximal upregulation of mTOR signaling pathway-related gene expression, increased phosphorylation of downstream proteins, and highest α-casein production—is achieved when valine, lysine, and threonine are supplemented at concentrations of 36.114 mmol/L, 9.027 mmol/L, and 4.602 mmol/L, respectively, corresponding to a molar ratio of 8:2:1. Furthermore, pharmacological inhibition of mTOR phosphorylation robustly attenuates α-casein synthesis, thereby reaffirming the critical role of the mTOR signaling pathway in mediating the stimulatory effects of this optimal amino acid combination on protein synthesis in MAC-T cells.

## Data Availability

We confirm that our experimental data are accurate, which supports the results and conclusions of this study.
